# Reproduction of contagious caprine pleuropneumonia reveals the ability of convalescent sera to reduce hydrogen peroxide production in vitro

**DOI:** 10.1186/s13567-019-0628-0

**Published:** 2019-02-08

**Authors:** Anne Liljander, Flavio Sacchini, Michael H. Stoffel, Elise Schieck, Nadine Stokar-Regenscheit, Fabien Labroussaa, Martin Heller, Jeremy Salt, Joachim Frey, Laurent Falquet, Danny Goovaerts, Joerg Jores

**Affiliations:** 1 International Livestock Research Institute, Box 30709, Nairobi, 00100 Kenya; 2 Institute of Veterinary Bacteriology, Vetsuisse Faculty, University of Bern, Länggass-Str. 122, Postfach, 3001 Bern, Switzerland; 3 Division of Veterinary Anatomy, Vetsuisse Faculty, University of Bern, Länggass-Str. 120, Postfach, 3001 Bern, Switzerland; 4 Institute of Animal Pathology (COMPATH), Vetsuisse Faculty, University of Bern, Länggass-Str. 122, Postfach, 3001 Bern, Switzerland; 5 Friedrich-Loeffler-Institute-Federal Research Institute for Animal Health, Naumburger Str. 96a, 07743 Jena, Germany; 6 GALVmed, Doherty Building, Pentlands Science Park, Bush Loan, Penicuik, Edinburgh, EH26 0PZ Scotland UK; 7 Division of Biochemistry, Department of Biology, University of Fribourg and Swiss Institute of Bioinformatics, Chemin du Musée 18, 1700 Fribourg, Switzerland; 8 Present Address: Istituto Zooprofilattico Sperimentale dell’Abruzzo e del Molise “G. Caporale”, via Campo Boario, 64100 Teramo, Italy

## Abstract

Contagious caprine pleuropneumonia (CCPP), caused by *Mycoplasma capricolum* subsp. *capripneumoniae* is a severe disease widespread in Africa and Asia. Limited knowledge is available on the pathogenesis of this organism, mainly due to the lack of a robust in vivo challenge model and the means to do site-directed mutagenesis. This work describes the establishment of a novel caprine challenge model for CCPP that resulted in 100% morbidity using a combination of repeated intranasal spray infection followed by a single transtracheal infection employing the recent Kenyan outbreak strain ILRI181. Diseased animals displayed CCPP-related pathology and the bacteria could subsequently be isolated from pleural exudates and lung tissues in concentrations of up to 10^9^ bacteria per mL as well as in the trachea using immunohistochemistry. Reannotation of the genome sequence of ILRI181 and F38^T^ revealed the existence of genes encoding the complete glycerol uptake and metabolic pathways involved in hydrogen peroxide (H_2_O_2_) production in the phylogenetically related pathogen *M. mycoides* subsp. *mycoides*. Furthermore, the expression of L-α-glycerophosphate oxidase (GlpO) in vivo was confirmed. In addition, the function of the glycerol metabolism was verified by measurement of production of H_2_O_2_ in medium containing physiological serum concentrations of glycerol. Peroxide production could be inhibited with serum from convalescent animals. These results will pave the way for a better understanding of host–pathogen interactions during CCPP and subsequent vaccine development.

**Electronic supplementary material:**

The online version of this article (10.1186/s13567-019-0628-0) contains supplementary material, which is available to authorized users.

## Introduction

**Figure 1:**
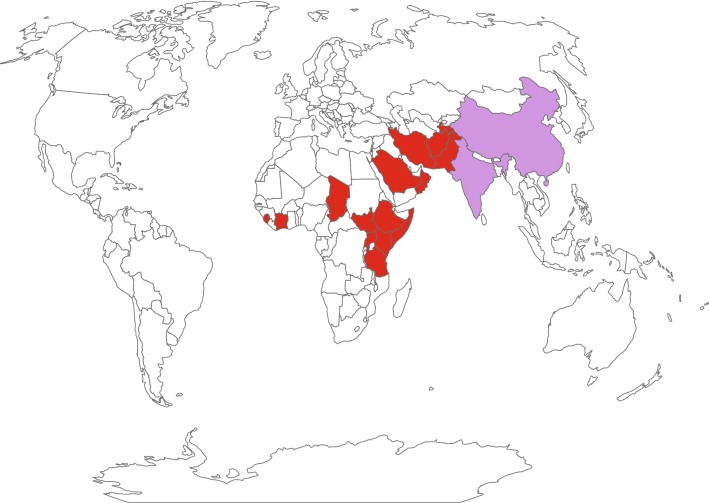
**Presence of contagious caprine pleuropneumonia from 2014 to 2018 based on the World Animal Health Information Database.** Countries displayed in red have the disease present and countries displayed in purple have the disease limited to one or more zones.

Contagious caprine pleuropneumonia (CCPP) is an important livestock disease that is widespread in the Middle East, Asia and Africa. Infection of goats with the causative agent *Mycoplasma capricolum* subsp. *capripneumoniae* (*Mccp*) causes pneumonia with respiratory symptoms that may progress into a lethal, generalized acute pleuropneumonia or to a chronic form with milder clinical signs and restricted pathomorphological lesions [1]. The infection is acquired through inhalation of contaminated droplets [2] and can cause morbidities and mortalities up to 100% and 80%, respectively [3]. Transmission of *M. capricolum* subsp. *capripneumoniae* to wild ungulates such as Arabian Oryx and Tibetan antelope has also been reported [4, 5]. Despite the fact that CCPP is on the list of diseases notifiable to the World Organization for Animal Health (OIE), only a few countries reported outbreaks between 2014 and 2018 (Figure 1). This might be due to lack of disease awareness, declining public funds to conduct surveillance and monitoring, suboptimal diagnostics and a possible misperception of CCPP symptoms with other respiratory diseases such as “peste des petits ruminants” (PPR) or *Pasteurella* spp. infections [6, 7]. During the last century, a bacterin-type of vaccine, co-formulating *M. capricolum* subsp. *capripneumoniae* type strain F38^T^ and saponin was developed for disease control in domestic goats [8]. Despite inducing immunity for up to 1 year, the use of a bactericidal adjuvant prohibits the inclusion of this vaccine in a combinatorial formula with live attenuated vaccines against additional caprine diseases such as PPR and capripox. The development of an efficacious vaccine formula against CCPP requires a robust and reproducible experimental challenge model [9]. Past infection models for CCPP include in-contact challenge [10–12], endobronchial inoculation [3, 12, 13] and intratracheal administration [10]. Although mimicking the natural disease transmission, in-contact experiments are often time-consuming, require a large number of animals and are difficult to standardize. The latter two infection methods are technically challenging and can result in different pathomorphological outcomes ranging from the absence of any clinical or pathomorphological changes to severe clinical disease and pathology [10, 13]. Consequently, an improved model to reproduce CCPP would not only enable vaccine efficacy studies but also foster in vivo studies that provide insights regarding the molecular mechanisms associated with pathogenicity and the virulence traits involved. The factors driving host–pathogen interactions in *Mccp* are not well understood. Hydrogen peroxide production, which is a candidate pathogenicity mechanism in many pathogenic *Mycoplasma* [14] has not investigated in *Mccp* yet.

Here we describe the establishment of a novel in vivo challenge model for CCPP that will pave the way for future vaccine development and vaccine efficacy studies. We expect this novel model to enable the research community to decipher CCPP-pathogenicity mechanisms and to identify virulence traits in *Mccp*. In addition, we report on the functionality of the enzymatic pathway leading to hydrogen peroxide production in *M. capricolum* subsp. *capripneumoniae*.

## Materials and methods

### *M. capricolum* subsp. *capripneumoniae* culture conditions

*Mycoplasma capricolum* subsp. *capripneumoniae* ILRI181 was isolated during a recent CCPP outbreak in Kenya [15], while type strain F38^T^ [3] was kindly supplied by the African Union Pan African Veterinary Vaccine Centre (AU-PANVAC), Ethiopia. Both strains were cultured in mycoplasma liquid medium containing a phenol-red pH indicator (Mycoplasma Experience Ltd, UK) at 37 °C under static conditions.

For the experimental challenges, *M. capricolum* subsp. *capripneumoniae* ILRI181 (2^nd^ passage) was cultured as described above to early logarithmic phase for 24–48 h (pH ≥ 6.8), aliquoted and stored in liquid nitrogen until further use. The infectious dose (color changing units [CCU/mL]) was determined by serial dilutions of two frozen aliquots including two technical replicates. The dilutions were incubated for 7 days. A color change from red to orange/yellow was considered as growth and the CCU/mL were determined [7].

### Production of polyclonal anti-*M. capricolum* subsp. *capripneumoniae* antibodies

The polyclonal antibodies were custom developed by BioGenes GmbH, Germany. In brief, rabbits were immunized intramuscularly with 200 µg heat-killed (100 °C for 10 min) *M. capricolum* subsp. *capripneumoniae* F38^T^ mixed with BioGenes adjuvants. Animals were boosted with the same total antigen/adjuvant formula on day 7 (100 µg), 14 (50 µg), 49 (50 µg), 63 (50 µg) and on day 70 (50 µg) post initial immunization. Polyclonal serum was harvested on day 77 post-immunization. For preservation purposes, thimerosal was added to the sera to a final concentration of 0.02%. Samples were stored at −20 °C until further use.

### Reannotation of the *M. capricolum* subsp. *capripneumoniae* ILRI181 and F38^T^ genome

The genomes of *M. capricolum* subsp. *capripneumoniae* ILRI181 and F38^T^ were reannotated (GenBank accession LN515399.1 and LN515398.1) using the Prokka pipeline [16] employing the previous draft genome [15], *M. mycoides* subsp. *mycoides* strain Afadé (GenBank accession LAEX00000000), type strain PG1 (GenBank accession NC_005364.2) and UniProtKB as additional databases.

### Phylogenetic analysis of l-α-glycerophosphate oxidase (GlpO) among members of the “*M. mycoides* cluster”

Amino acid sequences of GlpO from *M. capricolum* subsp. *capripneumoniae* ILRI181 and F38^T^ [15], 1601 [17], 9231-Abomsa [18], *M. mycoides* subsp. *mycoides* Afadé, B237 [19] PG1 [20], T1/44 [21] and Gladysdale [22], *M. leachii* PG50^T^ [22], *M. capricolum* subsp. *capricolum* ATCC27343^T^ (GenBank accession NC_007633) and *M. feriruminatoris* G5847^T^ [23, 24] (used as an outgroup) were retrieved from GenBank. Multiple alignments were generated using MUSCLE [25], curated from unreliable sites with Gblocks [26] and the phylogenetic tree was constructed by the Maximum Likelihood method using PhyML [27].

### In silico analysis of the l-α-glycerophosphate oxidase (GlpO) among members of the “*M. mycoides* cluster”

Amino acid sequences from all the selected member of the *Spiroplasma* phylogenetic group were retrieved as described above. Cellular localization of each individual GlpO was predicted using PSORTb [28] using the advanced Gram stain setting “negative without outer membrane” dedicated to analyzing *Mycoplasma* spp. organisms. Transmembrane helices and signal peptide cleavage predictions have been done using Phobius [29] and SignalP [30], respectively. SignalP prediction was done with “Gram-positive bacteria” as the organism group setting, as mycoplasmas lack the type I signal peptidase of Gram-negative bacteria used by the software to detect standard signal peptides for this group.

### In silico analysis of the flavin-adenine-dinucleotide (FAD)-binding site of GlpO

Amino acid sequences from the FAD-binding site of GlpO from *M. capricolum* subsp. *capripneumoniae* ILRI181 and l-2-hydroxyglutarate dehydrogenase (L2HDH) from several species e.g. bovine, goat, rabbit and mouse were obtained from various public databases; ENSEMBL [31], UniProtKB [32] and RCSB [33]. The sequence alignment was performed in MyHits [34] with MAFFT [35] defaults and viewed with Jalview [36]. The phylogenetic tree was calculated in Jalview with neighbor-joining method using BLOSUM62 distance. The 3D images were calculated in Chimera [37, 38] by aligning the structure of GlpO from *Escherichia coli* (2QCU) with GlpO peptide (DICIIGGGIIG) from *M. capricolum* subsp. *capripneumoniae* ILRI181.

### Experimental challenge model

#### Experimental animals

The sample size (*n* = 10 animals) was determined based on an expected morbidity of 80% induced by *M. capricolum* subsp. *capripneumoniae* ILRI181. We can thus ensure that with a 95% confidence the true morbidity is at least 55% (lowest reasonable threshold) if we observe 80% morbidity with 10 animals, using a 1-sample 2-sided exact calculation.

Ten outbred male goats (*Capra aegagrus hircus*), 1–2 years of age, weighing between 12.5 and 30.5 kg, were utilized in this study. The animals were randomly selected from the ILRI ranch in Kapiti (a CCPP-free region in Kenya), had not previously been vaccinated against CCPP and were seronegative to *M. capricolum* subsp. *capripneumoniae* prior to challenge. The animals were vaccinated against foot and mouth disease (FOTIVAX™, Kevevapi, Kenya) at the ILRI ranch on −44 days post-infection (dpi), transferred to the ILRI campus in Nairobi −30 dpi and kept in quarantine until −7 dpi. During the quarantine all animals were treated once for ectoparasites using Chlorpyrifos (500 g/L)/Cypermethrin 50 g/L (Duodip 55% Norbrook Kenya Ltd, Kenya) and twice for helminths, −23 dpi and −2 dpi using Levamisole Hydrochloride 3.0% w/v and oxyclozanide 6.0% w/v (Levafas Drench, Norbrook Kenya Ltd, Kenya) and Ivermectin 1% w/v (Noromectin, Norbrook, Kenya Ltd, Kenya) respectively, according to manufacturer’s recommendations. All animals were then vaccinated against enterotoxaemia (Jovaclost T, Jovac, Jordan), sheep and goat pox (S&G Pox™, Kevevapi, Kenya), foot and mouth disease (FOTIVAX™, Kevevapi, Kenya), PPR (Pestivax, Kevevapi, Kenya) and anthrax & blackleg (Blanthax vaccine, Cooper, Kenya) on −22 dpi, −16 dpi and −9 dpi, respectively. One week before the experimental infection, 10 goats were transferred to the animal biosafety level two (ABSL2) facility where they were all housed together for the remainder of the study. The animals were allowed to move freely within the ABSL2 room (28 m^2^ with sawdust bedding), had water, hay, and mineral lick ad libitum and received a portion of pellets in the morning (after the clinical examination) and in the afternoon. The animals were monitored twice daily (and on additional time points as needed) by a veterinarian and any medical concerns were addressed immediately. The study was conducted with animal welfare a high priority. Additionally, we included a mock-infected control group of three female and two male goats (CM233, CM251, CM253, CM260 and CM261) derived from the same population as the other animals and treated as described above.

#### Study design and experimental procedures

Ten goats were infected twice intranasally on two consecutive days (0 and 1 dpi) and once transtracheal by needle puncture, 5–10 cm caudal to the larynx (4 dpi). For the intranasal aerosol infection, thawed *M. capricolum* subsp. *capripneumoniae* ILRI181 liquid cultures (10^8^ CCU/mL), prepared as described above, were aspirated into a 1 mL syringe with attached atomizer (MAD Nasal™ Intranasal Mucosal Atomization Device, Teleflex^®^, UK) and each animal received 1 mL (500 µL/nostril) per infection. The final transtracheal infection was performed by administering 1 mL of culture (10^8^ CCU) followed by flushing with 5 mL of sterile phosphate buffered saline (PBS). Infectious material from all three challenges was serially diluted as described above to confirm the CCU. In addition to assessing behavior and appetite, the animals were closely monitored for any adverse reactions (at the site of nasal and transtracheal administration) and clinical signs of infection. Rectal temperature, blood oxygen saturation (measured at ear level), heart rate and breathing frequency were measured using a M750 digital thermometer (GLA Agriculture Electronics, USA), VE H100B Veterinary Pulse Oximeter (Edan, USA) and a stethoscope Classic II (Littmann, USA), respectively. Blood samples (taken by jugular vein puncture) and nasal swabs were taken twice per week starting 3 days before infection. The weight (kg) of the animals was measured once per week or daily at signs of clinical disease. Animals showing either a rectal temperature > 40.5 °C for > 3 consecutive days, signs of moderate to severe pain or distress, weight loss > 10% within 7 days or a breathing frequency of > 50/min for > 3 days were euthanized via intravenous injection of Sodium Pentobarbitone [220 mg/mL, Eutha-naze Injection, Bayer (Pty) Ltd Animal Health Div, South Africa] at a dosage of 100 mg/kg body weight. The remaining animals were euthanized 31 days post-infection (dpi). The mock-infected group was treated as above but received Mycoplasma medium without infectious agent. The mock-infected group was euthanized 35 dpi.

#### Hematological parameters

EDTA blood from *Mccp*-infected animals was used to measure the white blood cell (WBC) and red blood cell (RBC) count using the Celltac α MEK-6450 (Nihon Kohden, Japan). For caprine blood samples the parameters in the settings menu were adjusted as follows: WBC sensitivity = 10, WBC threshold = 7, RBC sensitivity = 15, RBC threshold: 3, RBC AUTO = OFF, PLT threshold = 5. The blood samples were thoroughly mixed with an equal volume of buffer (ISOTONAC 3, Nihon Kohden, Japan) prior to measurement. Provided values for WBCs, RBCs, HGB (hemoglobin) and HCT (hematocrit) were doubled prior to analysis to account for the buffer dilution. The analysis was carried out using the QP-821V Data Management Software LITE (Nihon Kohden, Japan).

#### Post-mortem analysis

Extensive post-mortem examinations were performed according to standard procedures [39]. Urine samples (1–2 mL per animal) taken at necropsy via needle puncture of the bladder were stored at −80 °C. When available, pleural fluid and lung juice (collected from a lung cut section) were collected and stored at −80 °C until further use. Fresh tissue samples of trachea and lung (different gross pathological lesions and unremarkable areas), heart, intestine, liver, kidney, spleen and several lymph nodes (retropharyngeal, prescapular, tracheal, peribronchial, mediastinal and mesenteric) were immediately fixed in a ready to use zinc formalin fixative solution (Sigma-Aldrich, USA) for 48 h at room temperature, transferred into 10% buffered formalin and subsequently embedded in paraffin for histological analysis.

#### Histology and immunohistochemistry

Paraffin-embedded tissue sections of 4 µm in thickness were stained with hematoxylin and eosin (HE) for histopathological evaluation. For immunohistochemistry (IHC), 4 μm sections were mounted on positively charged glass slides (Superfrost^®^plus, Thermo Scientific, Germany). After deparaffinization, rehydration and antigen retrieval (95 °C for 30 min), rabbit anti-*M. capricolum* subsp. *capripneumoniae* (used at 1:3000 dilution) stains were performed using an automated immunostainer Leica Bond RX (Leica Biosystems, Switzerland). Visualization was facilitated using the Bond Polymer Refine Detection kit (Leica Biosystems, Switzerland) according to the manufacturer’s instructions.

#### Serology

Antibody titers against *M. capricolum* subsp. *capripneumoniae* were determined in sequential serum samples using the IDEXX CCPP Ab Test (IDEXX, France) [6] according to the manufacturer’s instructions.

### Quantification of the hydrogen peroxide production (H_2_O_2_) of *M. capricolum* subsp. *capripneumoniae* in vitro and testing of the potential inhibitory effect by convalescent goat serum

To measure the production of hydrogen peroxide, *M. capricolum* subsp. *capripneumoniae* ILRI181 was grown as described above for about 26 h to end-exponential growth, when pH ≥ 6.9 and a density of approximately 10^7^–10^8^ CCU/mL was reached. Triplicate 1 mL cultures were taken for DNA extraction and another 1 mL aliquot for use in the hydrogen peroxide assay. DNA was extracted as described previously [40] and concentrations were measured using a Nanodrop 2000c spectrophotometer (Thermo Scientific, USA). For the peroxide assay, culture aliquots were centrifuged at 8000 × *g* for 10 min at 4 °C, washed once in cold PBS (4 °C, pH 7.3), centrifuged again and resuspended in pre-warmed PBS (37 °C). The suspensions were subsequently incubated at 37 °C for 1 h with serum from convalescent goats (decomplemented [56 °C for 30 min] and diluted 1:20, collected on −3 and 31 dpi), prior to centrifugation at 8000 ×* g* for 10 min at 37 °C, washed twice in pre-warmed PBS and resuspended in pre-warmed reaction buffer (provided in the kit described below). To induce H_2_O_2_ production, glycerol was added to the suspensions at a final concentration of 100 µM (the physiological concentration in caprine serum). Neat suspensions of ILRI181 only, with and without glycerol were included as a positive and negative control respectively. The production of H_2_O_2_ was measured using the Amplex Red Hydrogen Peroxide/Peroxidase Assay Kit (Life Technologies, UK) according to manufacturer’s instructions. Briefly, 50 μL of each sample was gently mixed with 50 μL of the Amplex Red reagent in opaque black 96-well plates (Costar^®^, Corning Incorporated, USA). Fluorescence was measured after a 30 min incubation using a Synergy HT microplate reader (BioTek, US) with excitation and emission set at 530 nm and 590 nm, respectively. A standard curve for H_2_O_2_ concentrations (provided in the kit) was included in the assay. Four technical replicates were performed for each sample and the assay repeated three times. The results were normalized against the DNA concentration. To enable a paired t-test comparing all pre- and post-infection samples, a 2-way ANOVA was initially used to check for differences in the change in response, pre- vs. post-infection, between animals (*n* = 4) and runs (times the experiment was repeated, *n* = 3).

### Western blot analysis for in vivo detection of GlpO

Western blot analyses were performed as described previously [41] but with the following modifications. Pleural fluid samples (1 mL) were centrifuged at 21 130 × *g* for 20 min prior to resuspension in PBS, lysis (99 °C for 10 min) and separation by 12% SDS PAGE. Gels were stained with Coomassie or transferred to nitrocellulose membranes (GE Healthcare Life Science) for Western blots. The membranes were blocked with 5% skimmed milk overnight at 4 °C prior to incubation with the primary antibody (rabbit IgG anti-GlpO), used at 1:100 dilution [42], for 1 h at room temperature. The membranes were subsequently incubated with horseradish peroxidase-conjugated secondary antibodies (anti-rabbit IgG [Sigma-Aldrich, USA] used at 1:1000 dilution) for 1 h at room temperature prior to adding the TMB substrate (Pierce™, Thermo Scientific, USA). *M. capricolum* subsp. *capripneumoniae* ILRI181, F38^T^ and *M. mycoides* subsp. *capri* GM12 cultures were included as positive controls.

### Bacteriology

For detection of live *M. capricolum* subsp. *capripneumoniae* serial dilutions (up to 10^−12^) were made from pleural fluid and lung juice in standard mycoplasma medium (Mycoplasma Experience Ltd, UK). The dilutions were cultured as described above and the CCU/mL were determined. When needed, lung tissue cultures were performed. In addition, nasal swabs were taken throughout the experiment and whole blood (taken prior to euthanasia), carpal joint fluid and urine collected at post-mortem were cultured in a 1:10 dilution. The presence of *M. capricolum* subsp. *capripneumoniae* was confirmed directly from culture material using a specific recombinase polymerase amplification (RPA) assay as described previously [7]. The pH of the urine was measured on thawed specimens using a total of three different pH test strips with overlapping range coverages (Fluka, Switzerland; Merck, Germany; Sigma, USA).

### Transmission electron microscopy

Regions of interest in lung tissue samples were selected based on pathological changes seen in corresponding paraffin sections and immunopositive staining with the anti-*M. capricolum* subsp. *capripneumoniae* antibody. Corresponding tissue areas were punched out from paraffin blocks, dewaxed in Neoclear (Merck, Switzerland) twice for 10 min, rehydrated through a descending ethanol series and transferred to 0.1 M cacodylate buffer (dimethylarsinic acid sodium salt trihydrate; Merck, Switzerland). Samples were post-fixed with 1% osmium tetroxide (OsO_4_, Polysciences, USA) in 0.1 M cacodylate buffer for 2 h at room temperature. After three washes in cacodylate buffer, the tissues were dehydrated again through an ascending ethanol series and transferred to acetone four times for 30 min each. Infiltration was carried out with mixtures of acetone/Epon (FLUKA, Switzerland) at ratios of 3:1 and 1:1 respectively, for 3 h each at room temperature. Samples were left in acetone/Epon at a ratio of 1/3 overnight at 4 °C, transferred to pure Epon and polymerized at 60 °C for 5 days. Semi-thin sections of 0.5 µm in thickness were stained with toluidine blue and used to localize areas of interest. Resin blocks were trimmed accordingly and ultrathin sections exhibiting silver interference were produced with diamond knives (Diatome, Switzerland) on a Reichert-Jung Ultracut E ultramicrotome (Leica, Switzerland). Ultrathin sections were collected on collodion-coated 200 mesh copper grids (Electron Microscopy Sciences, USA). Sections were then double-stained with 0.5% uranyl acetate (Sigma-Aldrich, Germany) for 30 min at 40 °C and 3% lead citrate (Leica, Switzerland) for 10 min at 20 °C in an Ultrastain^®^ (Leica, Austria) and examined in a Philips CM12 transmission electron microscope (FEI, Holland) at an accelerating voltage of 80 kV. Micrographs were captured with a Mega View III camera using the iTEM software version 5.2 (Olympus Soft Imaging Solutions GmbH, Germany).

## Results

### Reannotation of the genome of *M. capricolum* subsp. *capripneumoniae* ILRI181 and F38^T^

Reannotation of the genome of *M. capricolum* subsp. *capripneumoniae* ILRI181 and F38^T^ revealed the presence of gene *glpO* (locus tags: MCCPF38_00276 and MCCPILRI181_00272) upstream of *glpK* (encoding glycerol kinase). Gene *glpO* was originally miss-annotated as *lhgO* (2-hydroxyglutarate oxidase) by automatic genome annotation. Furthermore, the genes *gtsABC* (locus tags: MCCPF38_00541-3 and MCCPILRI181_00539-41) encoding the active ATP-dependent glycerol uptake system [43] were also identified.

### Phylogenetic relationship of GlpO among members of the “*M. mycoides* cluster”

**Figure 2:**
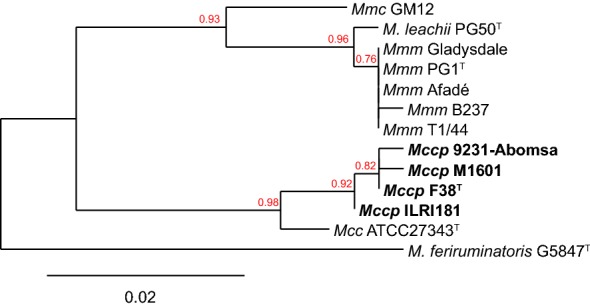
**Phylogenetic tree based on the GlpO amino acid sequences of “*M. mycoides*** **cluster” members.** The GlpO sequence of *M. feriruminatoris* was used as an outgroup. Bootstrap values are displayed. *Mcc*: *Mycoplasma capricolum* subsp. *capricolum*; *Mccp*: *Mycoplasma capricolum* subsp. *capripneumoniae*, *Mmc*: *Mycoplasma mycoides* subsp. *capri*; *Mmm*: *Mycoplasma mycoides* subsp. *mycoides.*

The phylogenetic relationship of GlpO among members of the “*M. mycoides* cluster” corresponds to the general phylogenetic relationship based on house-keeping genes [44] with the exception of GlpO from *M. leachii* which is related more to GlpO from *M. mycoides* subsp*. mycoides* than to *M. mycoides* subsp. *capri* (Figure 2). *M. mycoides* have been shown to produce peroxide. Therefore, we tested the ability of *Mccp* to produce peroxide in the presence of glycerol (see below).

### In silico characterization of GlpO among members of the “*M. mycoides* cluster”

All amino acid sequences available for this cluster were used to improve characterization of the GlpO cellular localization. No evidence of transmembrane helices was found using Phobius and no cleavage sites for signal peptides were identified. In addition, all sequences were predicted to be cytoplasmic with a score of 7.5 (out of 10).

### Flavin-adenine-dinucleotide (FAD)-binding site of GlpO

Since the goat proteome was not available at the time that this research was undertaken, we used the bovine proteome to search for homologies to the GlpO amino acid sequence. A small conserved region at the N-terminus of the protein was found, that displays similarities to a region of the mitochondrial l-2-hydroxyglutarate dehydrogenase (UniProtKB:A7MBI3) at the beginning of the FAD-binding domain in both proteins. Using ENSEMBL we identified orthologues to this L2HGDH gene in the goat genome and in other relevant species. A multiple sequence alignment was performed, and a single valine/isoleucine substitution was identified (Additional file 1). Furthermore, the *M. capricolum* subsp. *capripneumoniae* region was aligned to the 3D structure of *E. coli* GlpO (rcsb:2QCU) (Additional file 1) showing that the valine/isoleucine residue is located near to the FAD-binding site, although it does not look critical for FAD binding.

### Experimental in vivo challenge

#### Disease progression

**Figure 3:**
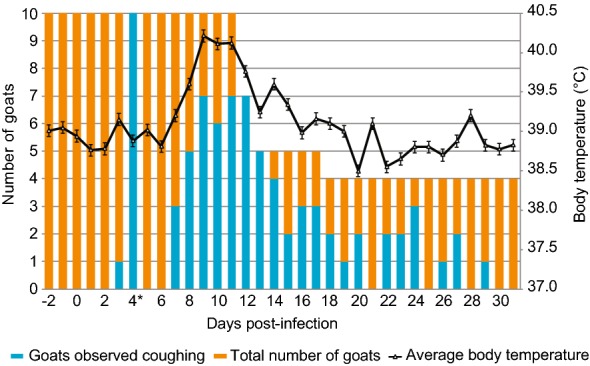
**Mean rectal temperature (SD) versus number of goats recorded coughing throughout the study period.** *Day of transtracheal infection. Error bars represent standard deviations.

Ten male goats (CK042, CM043, CM048, CM049, CM124, CM145, CM166, CM180, CM186, and CM189) were experimentally infected by an intranasal spray (0 and 1 dpi) and transtracheal injection (4 dpi) using live *M. capricolum* subsp. *capripneumoniae* ILRI181 (10^8^ CCU/mL/dose) (Additional file 2). Culturing of the remaining infectious material post-challenge confirmed titers of 10^8^ CCU/mL. No adverse reactions were seen at nasal or neck level throughout the study period. All animals developed pyrexia (defined as rectal temperatures > 39.5 °C) between 7 and 14 dpi with temperatures ranging from 39.6 to 41.4 °C (Additional file 3). The duration of fever varied between individual animals between 3 and 6 days. Concurrent with pyrexia, a majority of the animals developed a persistent cough (*n* = 7), with the highest number of coughing animals recorded between 9 and 12 dpi (Figure 3). Laborious and rapid breathing (breathing frequency of > 50/min) was documented in five animals (CK042, CM049, CM124, CM186, and CM189) coinciding with the peak of pyrexia (12 dpi, Additional file 3). Clinical disease was also associated with weight loss (Additional file 3) and six of ten animals experienced a drastic weight reduction of > 10% within 7 days. Additional sporadically observed clinical features included repeated sneezing, shivering, teeth grinding, ruffled/dull fur coat and diarrhea. Clinical disease was also associated with behavioral changes such as seeking solitude and standing with the head down. The above mentioned clinical features resembled textbook reports of CCPP [45]. Blood oxygen saturation always remained above 97% for all animals throughout the course of the study (data not shown). Six goats (CK042, CM049, CM124, CM180, CM186, and CM189) were euthanized between 12 and 17 dpi due to the severity of disease. The remaining animals, were euthanized at the end of the study period, 31 dpi. All five mock-infected goats (CM233, CM251, CM253, CM260 and CM261) were clinically healthy throughout the study period (Additional file 3).

#### Hematology

There was a marked increase in white blood cell count (WBC, 10^3^/μL) after 18 dpi (Additional file 4) in three out of four goats that got infected with *Mccp* and survived. The counts had however dropped to pre-infection level by 25 dpi. The red blood cell counts (RBC, 10^6^/μL) showed great fluctuations for all animals from 7 dpi until the end of the study (Additional file 4). As expected, the hemoglobin (HGB, g/dL) and hematocrit (HCT, %) followed the fluctuating pattern of the RBC counts (Additional file 4).

#### Pathological observations

**Figure 4:**
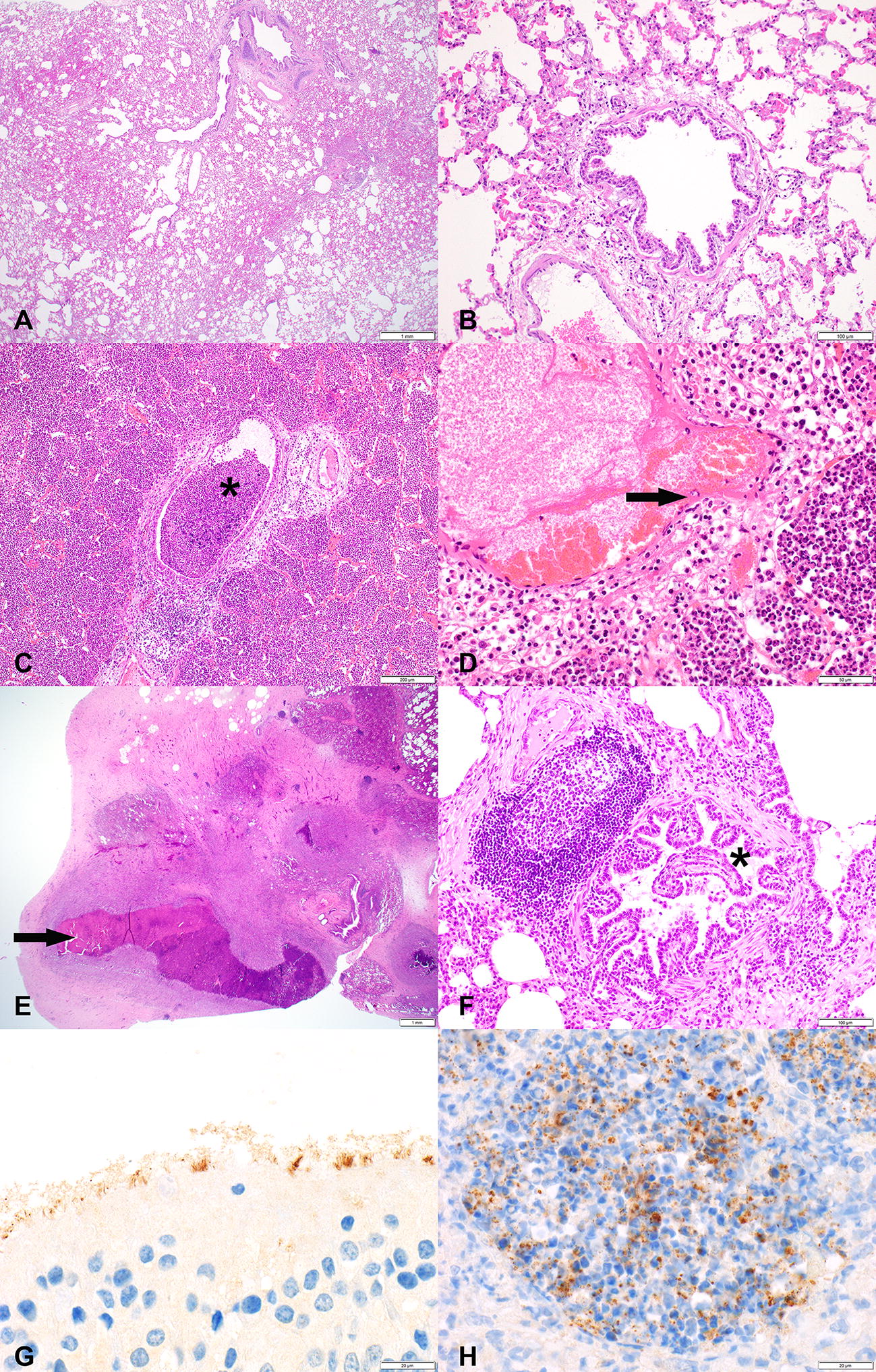
**Representative histopathological images (A–F) and immunohistochemistry (IHC) stainings (G–H) of caprine respiratory tissues.** Tissues are derived from goats experimentally infected with *Mycoplasma capricolum* subsp. *capripneumoniae* (**C**–**F**) and from a mock-infected control group (**A**, **B** no histopathological lesions present). **C**, **D** Lesions of the acute form of contagious caprine pleuropneumonia; airways filled with neutrophilic granulocytes (asterisk), edema, hemorrhage and fibrinoid degeneration and necrosis of vascular wall (arrow). **E**, **F** Lesions of the chronic form of CCPP; abscess formation with central coagulative necrosis and fibrous encapsulation (arrow) and the beginning of bronchiolitis obliterans in a bronchiole (clover). **G** *Mycoplasma capricolum* subsp. *capripneumoniae*-positive IHC staining on apical cell border of ciliated respiratory epithelial cells in the trachea. **H** *Mycoplasma capricolum* subsp. *capripneumoniae*-positive IHC staining in alveoli associated with neutrophilic granulocytes infiltration. Size standards are displayed in the lower right corner of each picture: **A** = 1 mm; **B** = 200 µm, **C** = 200 µm, **D** = 50 µm, **E** = 1 mm; **F** = 100 µm; **G** + **H **= 20 µm.

Post-mortem examination showed the presence of CCPP-typical macroscopic lesions in the lungs of all *Mccp*-infected animals (examples are given in Additional file 5), confirming successful experimental infection. Different pathological patterns of fibrinous bronchopneumonia were recorded; fibrinous adhesions, abundant fibrinous pleural effusion, lung consolidation, areas of coagulative necrosis and sequestra (Table 1). In six *Mccp*-infected animals the lesions affected both lungs, mainly involving apical, cardiac and accessory lobes and only rarely extending to the diaphragmatic lobes. The right lung was always affected. In animal CM180 with severe fibrinous bronchopneumonia, we also observed acute white infarcts in the kidney (Additional file 5). Histopathology revealed lesions of fibrinous bronchointerstitial pneumonia with pleuritis (pleuropneumonia) in the acute stage (Figures 4C and D). There were multiple areas of chronic lesions, i.e. abscess formation, pleural fibrosis and bronchiolitis obliterans (Figures 4E and F), leading to the overall diagnosis of chronic-active pleuropneumonia (Table 2). Immunohistochemistry for *M. capricolum* subsp. *capripneumoniae* showed strong positivity in the area of severe, acute inflammation, in close contact with or within alveolar neutrophils and macrophages in the alveoli (Figure 4H). A diffuse positive signal was detected within the ciliated epithelial cells of the trachea (Figure 4G). Typology, extension and lesion severity varied among the animals with severe lesions being more frequently observed in the animals that succumbed to disease than in recovering animals. No macroscopic or histopathological lesions were recorded in the mock-infected animals.

**Table 1:** **Characterization of pulmonary lesions in** ***Mycoplasma capricolum*** **subsp.** ***capripneumoniae*** **-infected goats**

Animal ID	Day of euthanasia	Lung portion affected	Consolidation	Coagulative necrosis	Sequestra (Ø in cm)	Pleural adhesions	Fibrinous adhesions	Pleural fluid (mL)
CK042	13	RA/C LC	Red/grey	+	−	−	+	68
CM043	31	RA/C/D LC	−	−	−	+	−	−
CM048	31	RA	Red	−	Multiple (0.3–0.5)	−	−	−
CM049	12	RA/C/D AL LA/C	Red/grey	+	−	−	+	100
CM124	12	RA/C	Red/grey	+	−	−	+	170
CM145	31	RA/C	−	−	1.0	+	−	3
CM166	31	RC	−	−	2.0	+	−	−
CM180	17	RA/C LA/C/D	Red/grey	+	−	−	+	150
CM186	12	RA/C/D LA/C/D	Red/grey	+	−	−	+	35
CM189	13	RA/C/D AL LA	Red/grey	+	−	−	+	30

Mock-infected animals are not displayed, since they did not have any lesions.

RA: right apical lobe, RC: right cardiac lobe, RD: right diaphragmatic lobe, AL: accessory lobe, LA: left apical lobe, LC: left cardiac lobe, LD: left diaphragmatic lobe.

**Table 2:** **Acute and chronic histopathology lesions observed in lung samples of** ***Mycoplasma capricolum*** **subsp.** ***capripneumoniae*** **-infected goats**

Animal ID	Purulent exudate^a^	Coagulative necrosis^a^	Fibrinoid degeneration and necrosis of vessels/vasculitis, thrombosis^a^	Distended interlobular septae by edema and fibrin^a^	Perivascular lymphocytic cuffing^a, b^	Pulmonary abscess^b^	Bronchiolitis obliterans^b^	Pleural fibrosis^b^	Stage of pneumonia^c^
CK042	+	+	+	+	+	−	−	−	A
CM043	+	+	−	−	+	−	+	+	CA
CM048	+	+	−	−	+	+	+	+	CA
CM049	+	+	+	+	+	−	−	−	A
CM124	+	+	+	+	−	−	−	+	CA
CM145	+	+	+	−	+	+	−	+	CA
CM166	+	+	+	+	+	+	+	+	CA
CM180	+	+	+	+	+	−	−	+	CA
CM186	+	+	+	+	+	−	−	+	CA
CM189	+	+	+	+	+	−	+	+	CA

Mock-infected animals are not displayed, since they did not show any lesions.

^a^Histopathological criteria for acute stage of pneumonia.

^b^Histopathological criteria for chronic stage of pneumonia.

^c^Stages according to time of inflammation and adjacent reaction and regeneration mechanisms: A: acute, CA: chronic-active.

### Serological responses to *M. capricolum* subsp. *capripneumoniae*

In the *Mccp*-infected group, seroconversion started from 11 dpi and by 14 dpi, all surviving animals had positive responses that remained above cutoff throughout the study period (Additional file 6). The six animals that were euthanized due to disease severity were serologically negative. The mock-infected goats remained seronegative for *Mccp* throughout the experiment (Additional file 6).

### Ability of the post-infection sera to block H_2_O_2_ production of *M. capricolum* subsp. *capripneumoniae* in vitro

**Figure 5:**
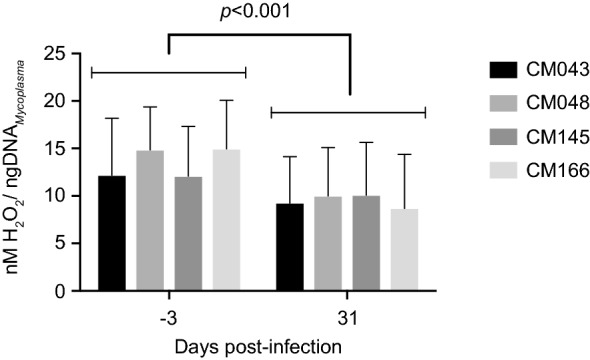
**Production of hydrogen peroxide by** ***M. capricolum*** **subsp.** ***capripneumoniae*** **ILRI181.** Pre-infection (left) and post-infection (right) sera from goats that recovered from a course of experimental contagious caprine pleuropneumonia were added to the medium. Error bars represent standard deviations from three biological replicates.

In the presence of caprine serum concentrations of glycerol (100 µM), *M. capricolum* subsp*. capripneumoniae* ILRI181 released on average 9.9 nM H_2_O_2_/ng DNA after 30 min of incubation (Figure 5). A significant reduction in the production was observed in the presence of post-infection sera as compared to pre-infection sera *p* < 0.001 (t11 = 5.17) (Figure 5). This corresponds to an average reduction of 4.022 nM H_2_O_2_/ng DNA (95% CI 2.31, 5.73). Minimal H_2_O_2_ release was observed in the control preparations containing mycoplasma and incubation buffer only.

### In vivo detection of GlpO

Western blot analysis of pleural fluid samples collected at post-mortem revealed a positive signal at a size of about 42–43 kDa in four of the seven included animals (Additional file 7) using the rabbit IgG anti-GlpO antibody [42]. Pure cultures of *M. capricolum* subsp. *capripneumoniae* ILRI181, F38 and *M. mycoides* subsp. *capri* GM12 had a similar band profile.

### Isolation of *M*. *capricolum* subsp. *capripneumoniae*

*Mycoplasma capricolum* subsp. *capripneumoniae* was isolated from pleural fluid and/or lung juice from all but one of the *Mccp*-infected animals with titers reaching 10^9^–10^10^ CCU/mL (Table 3). Goat CM043 showed minimal clinical symptoms, very mild pathological lesions and samples from this animal, including lung tissues, were negative. Furthermore, whole blood taken prior to euthanasia and urine collected at post-mortem from the ten *Mccp*-infected animals were all culture negative and only one of the carpal joint fluid cultures (from animal CM124) was positive. Biweekly nasal swabs were occasionally positive in the *Mccp*-infected animals; animals CK042, CM049, and CM124 had positive swabs on 11 dpi, while animal CM048 had positive swabs on 11 and 18 dpi. The urine from the six animals that were euthanized during the acute phase of the disease had an average pH of 6.4 (± 0.26) whereas that of the four animals that recovered from the acute stages and the mock-infected animals had an physiological average pH of 8.5 (± 0.2) (Additional file 8).

**Table 3:** **Results of culture and molecular identification of** ***M. capricolum*** **subsp.** ***capripneumoniae*** **from biological samples**

Animal ID	Pleural fluid		Lung juice	
	CCU/mL	RPA	CCU/mL	RPA
CK042^a^	10^6^–10^7^	Positive	10^9^	Positive
CM043	n/a	n/a	n/a	n/a
CM048	n/a	n/a	10^4^–10^5^	Positive
CM049^a^	10^2^–10^4^	Positive	n/a	Positive
CM124^a^	10^9^–10^10^	Positive	10^8^	Positive
CM145	Negative	Negative	10^3^	Positive
CM166	n/a	n/a	10^5^	Positive
CM180^a^	10^9^	Positive	10^9^–10^10^	Positive
CM186^a^	10^9^	Positive	10^6^	Positive
CM189^a^	10^2^–10^3^	Positive	10^9^	Positive

Mock-infected animals are not displayed, since *Mycoplasma capricolum* subsp. *capripneumoniae* was not detected in any of their specimens.

n/a: not analyzed, CCU/mL: color changing units per milliliter, RPA: recombinase polymerase amplification.

^a^Euthanized before 31 dpi.

### Transmission electron microscopy

**Figure 6:**
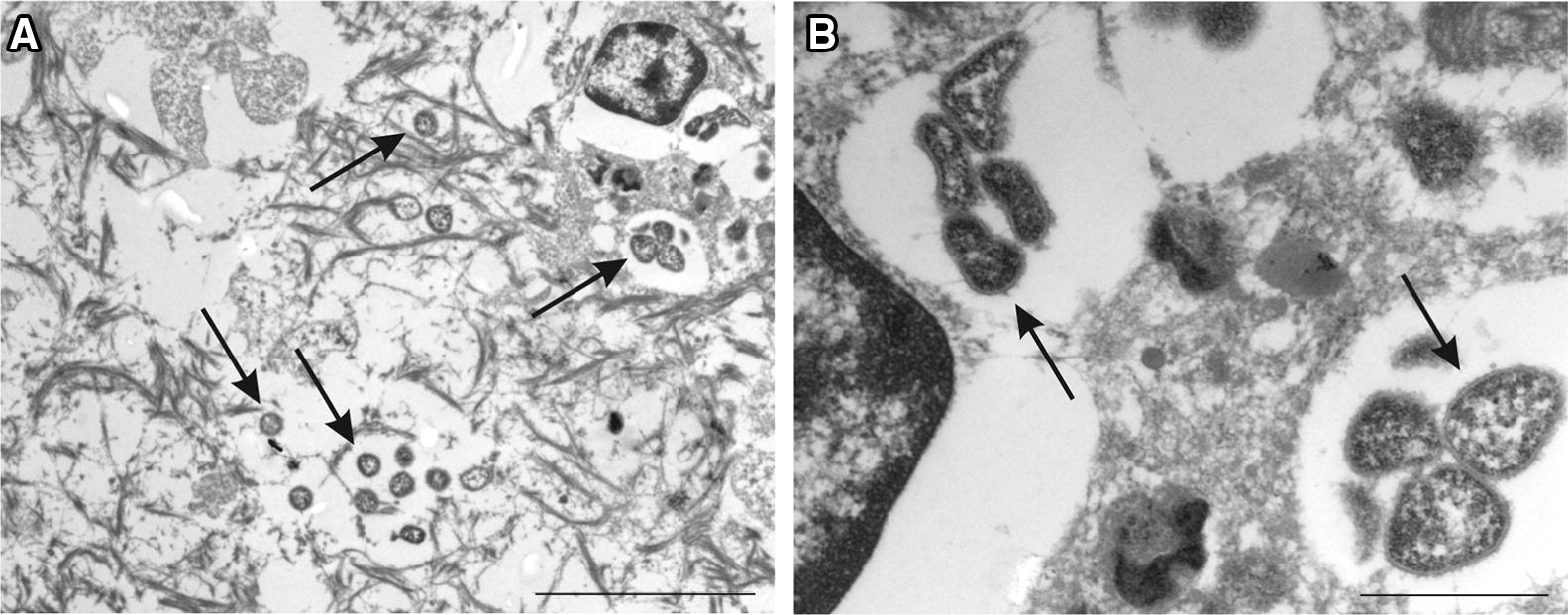
**Electron micrographs of mycoplasma cells in caprine lung tissue.** *Mycoplasma* cells are indicated by arrows. Scale bars: **A** (5 μm), **B** (1 μm).

In spite of the suboptimal tissue preservation and previous paraffin embedding, mycoplasmas were identified in electron micrographs of lung tissue based on morphological criteria such as their size (0.1–0.2 µm × 1–2 µm), pleomorphic shape and the single, trilayered membrane (Figures 6A and B).

## Discussion

Here we report on the development of a new caprine challenge model for contagious caprine pleuropneumonia (CCPP) that can be applied in resource-limited environments, common in many low and middle-income countries (LMIC). As repeated contact is required for the transmission of many mycoplasmal diseases the method developed here incorporates recurring exposure to the causative agent *M. capricolum* subsp. *capripneumoniae*. Ten male goats were experimentally infected via two intranasal spray applications followed by a total transtracheal deposit of 3 × 10^8^ color changing units (CCU). This approach was considered safe as no adverse reactions were seen at either site of inoculation. Moreover, to assure a defined inoculum of live mycoplasma, frozen aliquots (−80 °C) with a predetermined bacterial titer were used as already reported for *M. mycoides* subsp. *mycoides* [46]. The challenge dose used here is rather low compared to previous experimental *M. mycoides* subsp. *mycoides* and *M. capricolum* subsp. *capripneumoniae* infections where doses of 10^9^–10^10^ mycoplasma have been used [47–50], yet, morbidity and mortality rates of 100% and 60% respectively, were achieved. The inoculum applied in this experiment is however high compared to recent experimental *M. bovis* infections in calves where infection doses of 4 × 10^4^ CCU/animal were administered in aerosol chambers [51]. On the other hand, infectious doses based on color changing units (CCU) might not be 100% comparable between different mycoplasma species. Nevertheless, since we opted for a challenge model that can be easily applied in LMIC, we strongly believe that this challenge model is well suited since it does not rely on tailor-made equipment and can even be applied under field settings. While our novel challenge model resulted in 60% of the animals reaching the endpoint criteria, we did not alter the titer of the challenge dose, which retrospectively would have been desirable to determine a possible correlation of clinical disease, pathology and infectious dose. Nevertheless, all experimentally infected animals developed clinical signs, displaying elevated body temperatures (> 39.5°) with onset 7–14 dpi. Acute disease was characterized by high fever (> 40.5°, *n* = 8), a frequent and persistent cough (*n* = 7), rapid breathing (> 50/min, *n* = 5) and swift weight loss (*n* = 6). In addition, six animals had to be euthanized prior to the envisioned end of the study due to disease severity.

The attributes of virulence of strain ILRI181 resulting in this elevated pathogenicity remain unknown. However, the absence of a high number of passages and the relatively low numbers of generations that strain ILRI181 has been grown in axenic medium since its primary isolation might explain its superior pathogenicity compared to other strains [52], despite the overall clonal structure of the species [44, 53, 54]. True reproduction of infection was confirmed by the successful isolation of mycoplasmas from all but one animal, with high concentrations detected in lung juice and pleural fluid samples (Table 3) and the absence of clinical and pathomorphological changes in the five mock-infected animals. In addition, the presence of mycoplasmas in lung tissue was confirmed by immunohistochemistry (Figure 4). Unexpectedly, *M. capricolum* subsp*. capripneumoniae* were also detected in the trachea (Figure 4E), likely as a result of the mucociliary clearance, the expulsion of mycoplasma from the lung via coughing or from local colonization. Mucociliary colonization has previously been described for other mycoplasmas such as *M. hyopneumoniae* [55]. The colonization of the upper respiratory tract by *M. capricolum* subsp*. capripneumoniae* might explain the higher infectivity compared to *M. mycoides* subsp. *mycoides* [56], which has not been reported to colonize the trachea in vivo [46, 47]. Future studies should focus on investigating a potential colonization of the epithelial cells in the upper respiratory tract in order to characterize any local cytotoxicity.

The mechanism associated with the pathogenesis seen during CCPP remain largely unknown and common bacterial virulence factors have not been found in the mycoplasma genome [20]. Here, the presence of the candidate mycoplasma virulence factor, GlpO, the enzyme involved in the generation of H_2_O_2_ during the oxidation of glycerol-3-phosphate, was investigated [14, 57–59]. The *glpO* gene has so far not been found in the genome of *M. capricolum* subsp*. capripneumoniae* F38 and ILRI181 by automatic annotation [15]. However, our reannotation of the genome sequence of *M. capricolum* subsp. *capripneumoniae* did indeed reveal the presence of the genes encoding the biologicals capable of assimilating glycerol added to the media at physiological concentrations, and to metabolize it to dihydroxyacetone phosphate with the release of toxic H_2_O_2_. The in vivo expression of GlpO was also confirmed in pleural fluid from diseased animals (Additional file 7). The in vitro H_2_O_2_ production was significantly reduced, when *Mccp* cells were incubated with serum from convalescent goats, taken 31 dpi (Figure 5). It has however previously been shown that cattle immunized with recombinant GlpO from *M.* *mycoides* subsp. *mycoides* failed to generate neutralizing antibodies and succumbed to disease after subsequent challenge, this despite mounting GlpO-specific antibodies [60]. The inability to raise a neutralizing humoral immune response to GlpO was hypothesized to be due to the high similarity of the flavin-adenine-dinucleotide (FAD)-binding site, one of the main active sites of GlpO from *M. mycoides* subsp. *mycoides* to the bovine FAD-binding site, but not murine or rabbit l-2-hydroxyglutarate dehydrogenase (L2HDH) [60]. Indeed, the same single amino-acid substitution was also identified in goats (Additional file 1). The reduction in H_2_O_2_ seen between samples taken pre- and post-infection is indicative of the presence of antibodies inhibiting peroxide production. This is likely due to the fact that in the current study, goats were experimentally infected with live wild-type *M. capricolum* subsp. *capripneumoniae*, and hence are likely to have mounted antibodies against different epitopes of GlpO and against many other *Mycoplasma* components linked to the glycerol metabolism. However, following our in silico analysis on all the different GlpO amino acid sequences retrieved among the members of the “*M. mycoides* cluster”, we could not identify any transmembrane domains or signal peptides. Therefore, all sequences were predicted to be cytoplasmic. This is in agreement with wet laboratory data obtained for *M. pneumoniae* [57] and for *M. mycoides* [61, 62] but in contradiction with previous scanning electron microscopy photographs on *M. mycoides* subsp. *mycoides* [42]. A cytoplasmatic localization would support the inability to produce neutralizing antibodies against GlpO, since they simply cannot reach its target. Based on these results, we anticipate that mutagenesis of the key enzymes involved in glycerol metabolism might attenuate *M. capricolum* subsp. *capripneumoniae*. This will be tested in future studies that aim to develop a live vaccine against CCPP. In conclusion, the establishment of an easy to use challenge model will foster the scientific efforts towards a better understanding of CCPP, which not only has a high impact on goats but also on the life of many livestock-dependent people in LMIC.

## Additional files

**Additional file 1.** **In silico analysis of the flavin-adenine-dinucleotide (FAD)-binding site of L-α-glycerophosphate oxidase (GlpO).** A) Multiple sequence alignment of the L2HGDH peptide from several species colored with Jalview according to ClustalX schema. The sequence names are colored according to the tree split (vertical red bar). The fifth position of the alignment (red arrow) is suspected to distinguish hosts with “V” in L2HGDH in which the GlpO peptide would become immunogenic from non-responsive hosts with “I” in L2HGDH. B) Predicted structure model of the GlpO from *Escherichia coli* (2QCU) (light blue secondary structure) aligned to the immunogenic peptide from ILRI181 (DICIIGGGIIG) in orange. Note that the location of this peptide is in close neighborhood to the FAD co-factor (ball&stick model).

**Additional file 2.** **Experimental challenge of goats with** ***Mycoplasma capricolum*** **subsp.** ***capripneumoniae*** **ILRI181.** A) intranasal spray infection; covering one nostril at the time, the infectious material (500 μL/nostril) was administered through a syringe fixed with an atomizer, B) placement of the needle, 5 to 10 cm distal to the larynx, for the transtracheal administration, C), injection of the *Mycoplasma*-containing broth prior to flushing with sterile PBS. A successful administration was confirmed by subsequent coughing.

**Additional file 3.** **Body temperature, respiratory rate and body weight dynamics.** A: Clinical parameters measured from individual *Mccp*-infected animals. Top panel: body temperature, the lower dotted line indicates fever (39.5 °C), the upper dotted line indicates high fever (40.5 °C); Middle panel: breathing frequency per minute, the dotted line indicates a high breathing frequency of 50/min; Bottom panel: weight change in kg throughout the trial. B: Clinical parameters measured from individual *mock*-infected animals. Top panel: body temperature, the lower dotted line indicates fever (39.5 °C), the upper dotted line indicates high fever (40.5 °C); Middle panel: breathing frequency per minute, the dotted line indicates a high breathing frequency of 50/min; Bottom panel: weight change in kg throughout the trial.

**Additional file 4.** **Haematological parameters measured from individual** ***Mccp*-infected animals.** A) white blood cell count (10^3^/μL), B) red blood cell count (10^6^/μL), C) haemoglobin levels (g/dl) and D) haematocrit (%).

**Additional file 5.** **Macroscopic lesions observed during post-mortem.** A: thoracic cavity with yellow fibrin accumulated between the parietal and visceral pleura in the right lung, creating fibrinous adhesions between the lung and the chest wall; B: lung showing fibrinous pleuropneumonia with extended deposits of fibrin covering the pulmonary (right) surface; C: pneumonia affecting more than 60% of the right lung parenchyma (apical lobe, medium lobe and cranial part of diaphragmatic lobe); D: transverse section of lung, acute inflammation of the parenchyma with congestion and pulmonary edema; E: cut section of lung lobe with pneumonia. Parenchyma is firm showing areas varying from acute inflammation (reddish) to necrosis (grayish); F: lung sequestra; necrotic tissue is surrounded by a white fibrotic capsule; G: enlarged and hemorrhagic respiratory lymph nodes (mediastinal and peribronchial); H: fibrinous pleural exudate collected from the thorax cavity of a single goat; I: kidney infarct, hemorrhagic area is surrounded by a pale white zone.

**Additional file 6.** **Serological responses against** ***Mycoplasma capricolum*** **subsp.** ***capripneumoniae*** **measured by IDEXX CCPP Ab Test.** A) Table displaying individual serological responses of *Mccp*-infected animals. B) Antibody response (mean values) of euthanized vs surviving goats within the *Mccp*-infected group. C) Table displaying individual serological responses of mock-infected animals. All values are expressed as % of inhibition. Cutoff = 55%. Positive ≥ 55%.

**Additional file 7.** **In vivo detection of glycerol-3-phospate oxidase (GlpO, 42.6** **kDa).** Pleural fluid samples were separated by SDS-PAGE and were transferred to nitrocellulose membranes for subsequent immunoblot analysis using a polyclonal rabbit anti-GlpO antibody. The right side contains the loading control of the samples that have been used in the immunoblot on the left side (Coomassie stain). Positive controls were the strains *Mycoplasma capricolum* subsp. *capripneumoniae* F38^T^ and ILRI181 as well as *Mycoplasma mycoides* subsp. *capri* GM12 all expressing GlpO, negative controls were pleural fluids from animals CM189 and CM049 that contained very few *Mycoplasma*.

**Additional file 8.** **Summary table of urine pH taken at necropsy.**

## Abbreviations

ABSL2: animal biosafety level two facility
AU-PANVAC: African Union Pan African Veterinary Vaccine Centre
CBPP: contagious bovine pleuropneumonia
CCPP: contagious caprine pleuropneumonia
CCU: color changing units
dpi: days post-infection
FAD: flavin-adenine-dinucleotide
GlpO: l-α-glycerophosphate oxidase
H_2_O_2_: hydrogen peroxide
HCT: hematocrit
HE: hematoxylin and eosin
HGB: hemoglobin
IACUC: institutional animal care and use committee
IHC: immunohistochemistry
ILRI: International Livestock Research Institute
L2HDH: l-2-hydroxyglutarate dehydrogenase protein
LMIC: low and middle-income countries
OIE: World Organization for Animal Health
PBS: phosphate buffered saline
PPR: peste des petits ruminants
RBC: red blood cell
RPA: recombinase polymerase amplification
WBC: white blood cell
