# Supplementary figures and images for: Reproduction of contagious caprine pleuropneumonia reveals the ability of convalescent sera to reduce hydrogen peroxide production in vitro
Source: Vet Res. 2019 Feb 8;50:10. doi: 10.1186/s13567-019-0628-0 (PMC6368817; doi:10.1186/s13567-019-0628-0)

# A

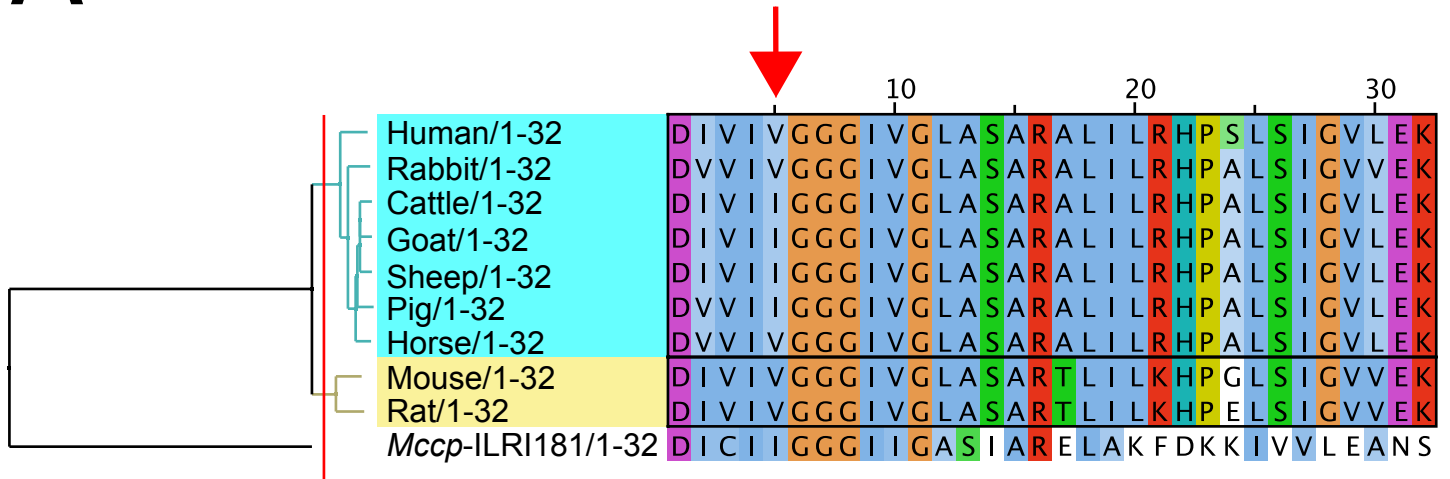

# B

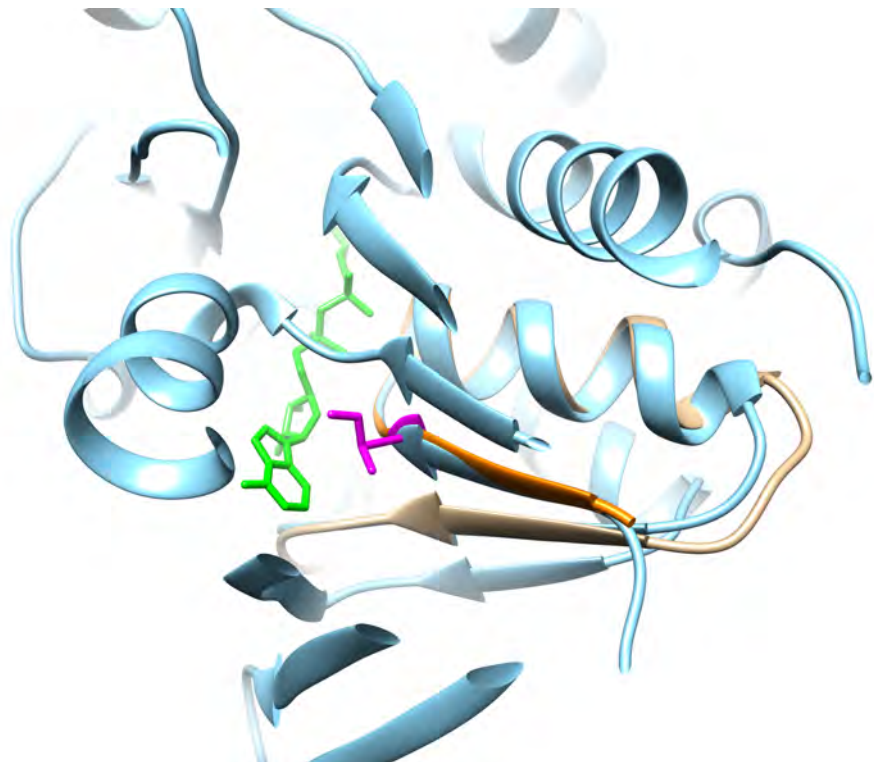

Supplement: Supplementary file 1 — Additional file 1. In silico analysis of the flavin-adenine-dinucleotide (FAD)-binding site of L-α-glycerophosphate oxidase (GlpO). A) Multiple sequence alignment of the L2HGDH peptide from several species colored with Jalview according to ClustalX schema. The sequence names are colored according to the tree split (vertical red bar). The fifth position of the alignment (red arrow) is suspected to distinguish hosts with “V” in L2HGDH in which the GlpO peptide would become immunogenic from non-responsive hosts with “I” in L2HGDH. B) Predicted structure model of the GlpO from Escherichia coli (2QCU) (light blue secondary structure) aligned to the immunogenic peptide from ILRI181 (DICIIGGGIIG) in orange. Note that the location of this peptide is in close neighborhood to the FAD co-factor (ball&stick model). [file 13567_2019_628_MOESM1_ESM.pdf]

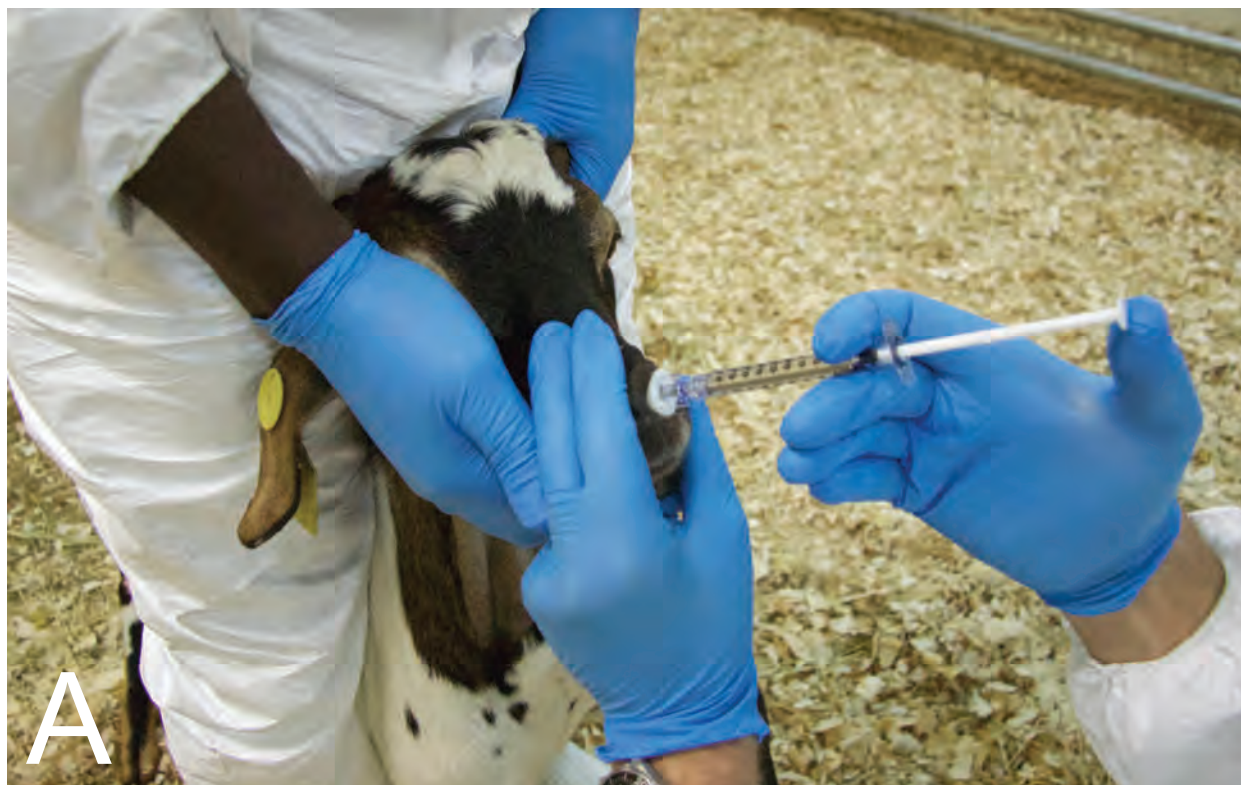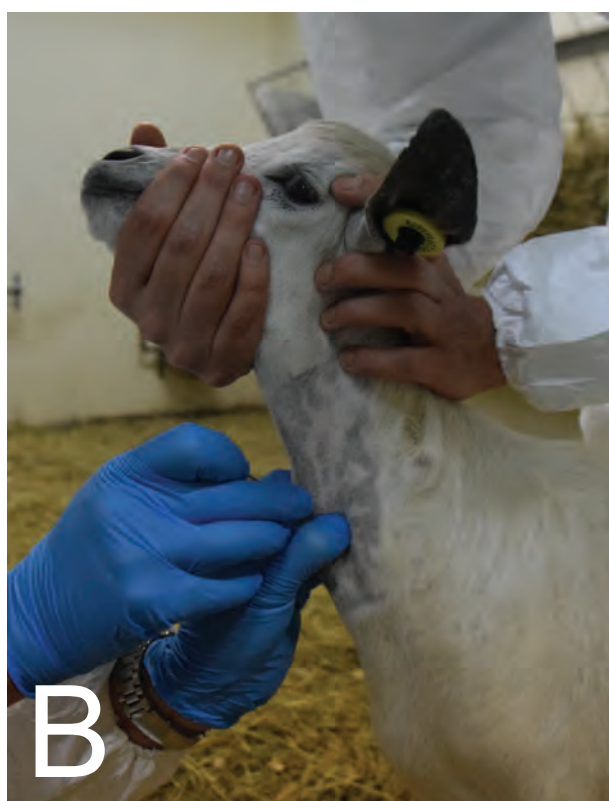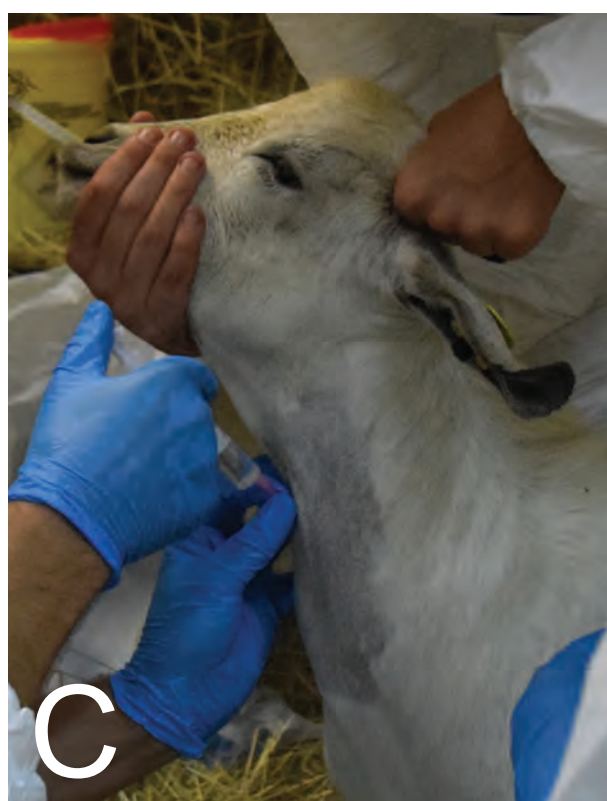

Supplement: Supplementary file 2 — Additional file 2. Experimental challenge of goats with Mycoplasma capricolum subsp. capripneumoniae ILRI181. A) intranasal spray infection; covering one nostril at the time, the infectious material (500 μL/nostril) was administered through a syringe fixed with an atomizer, B) placement of the needle, 5 to 10 cm distal to the larynx, for the transtracheal administration, C), injection of the Mycoplasma-containing broth prior to flushing with sterile PBS. A successful administration was confirmed by subsequent coughing. [file 13567_2019_628_MOESM2_ESM.pdf]

A

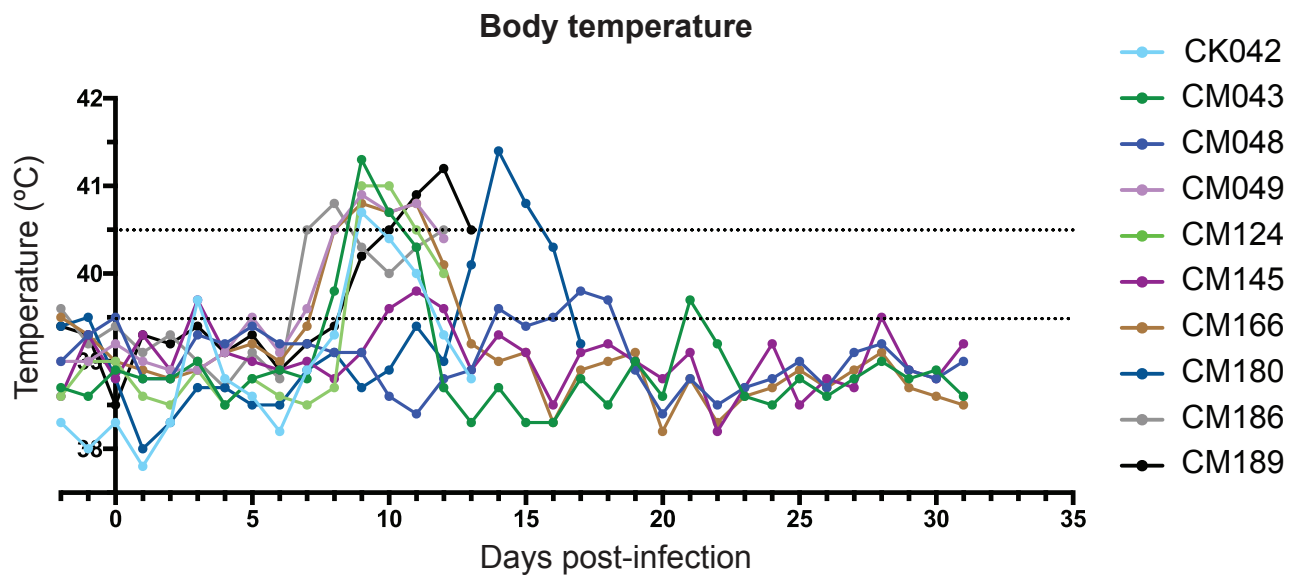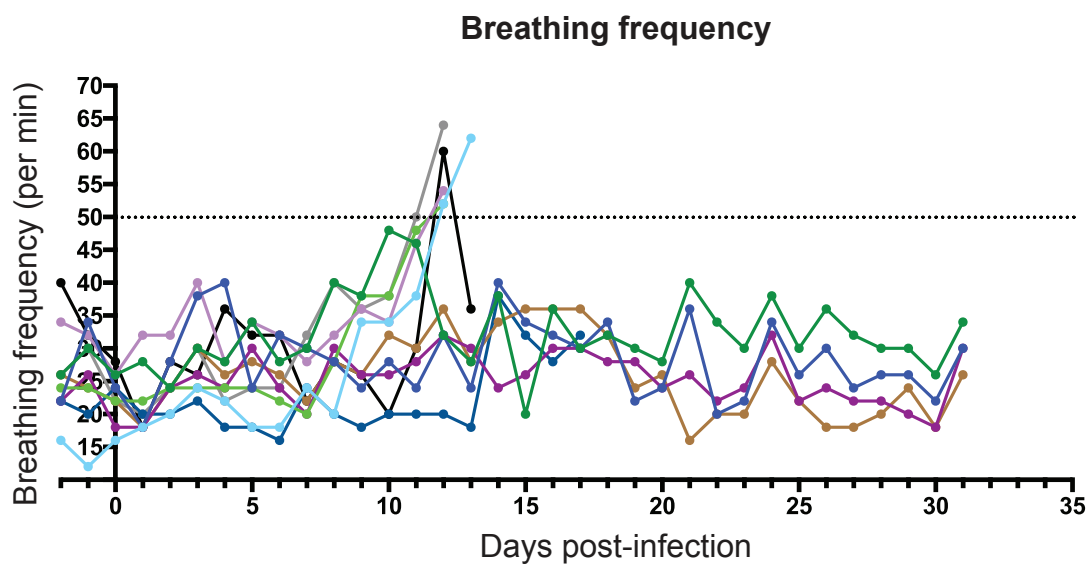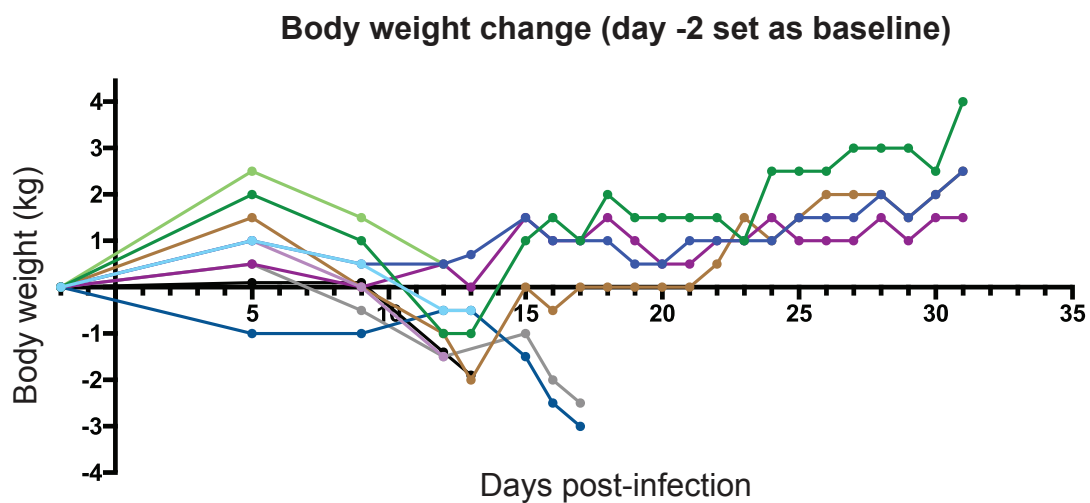

B

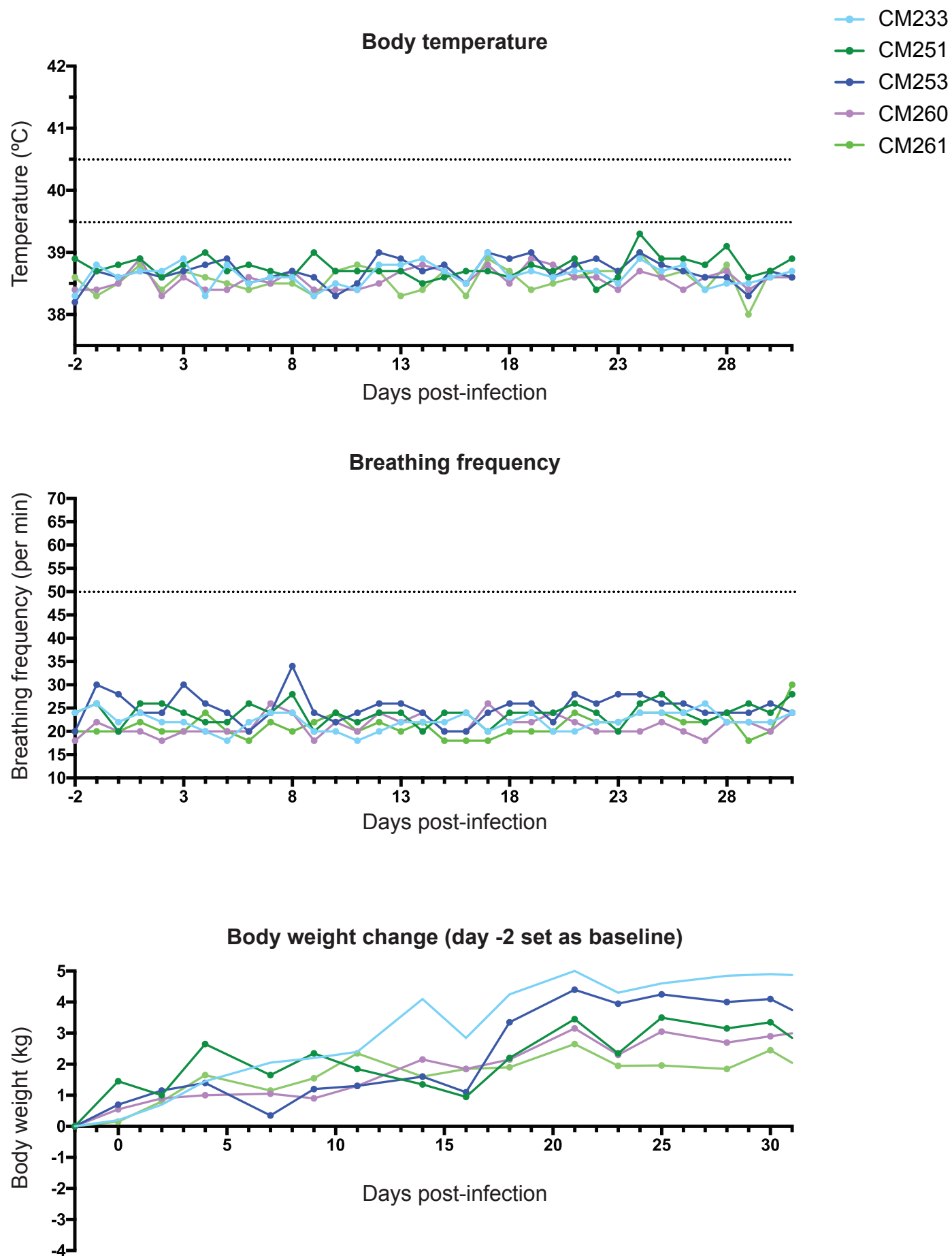

Supplement: Supplementary file 3 — Additional file 3. Body temperature, respiratory rate and body weight dynamics. A: Clinical parameters measured from individual Mccp-infected animals. Top panel: body temperature, the lower dotted line indicates fever (39.5 °C), the upper dotted line indicates high fever (40.5 °C); Middle panel: breathing frequency per minute, the dotted line indicates a high breathing frequency of 50/min; Bottom panel: weight change in kg throughout the trial. B: Clinical parameters measured from individual mock-infected animals. Top panel: body temperature, the lower dotted line indicates fever (39.5 °C), the upper dotted line indicates high fever (40.5 °C); Middle panel: breathing frequency per minute, the dotted line indicates a high breathing frequency of 50/min; Bottom panel: weight change in kg throughout the trial. [file 13567_2019_628_MOESM3_ESM.pdf]

A

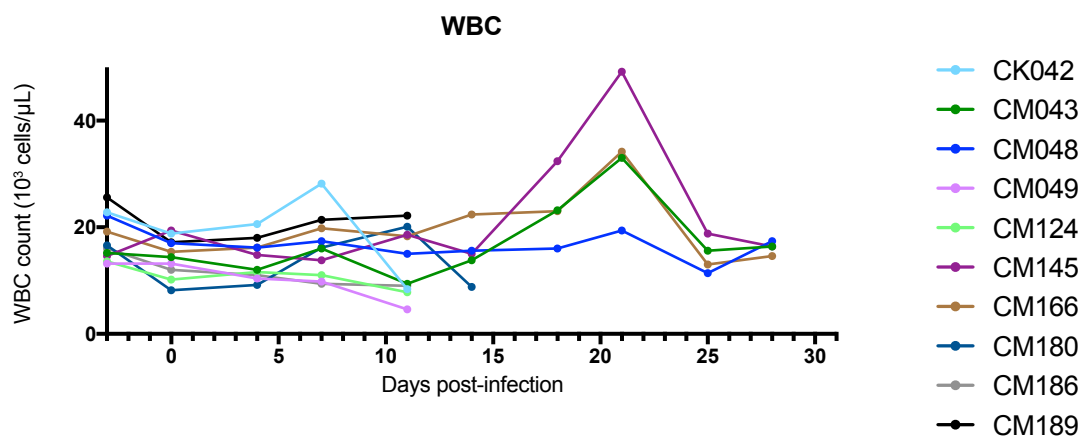

B

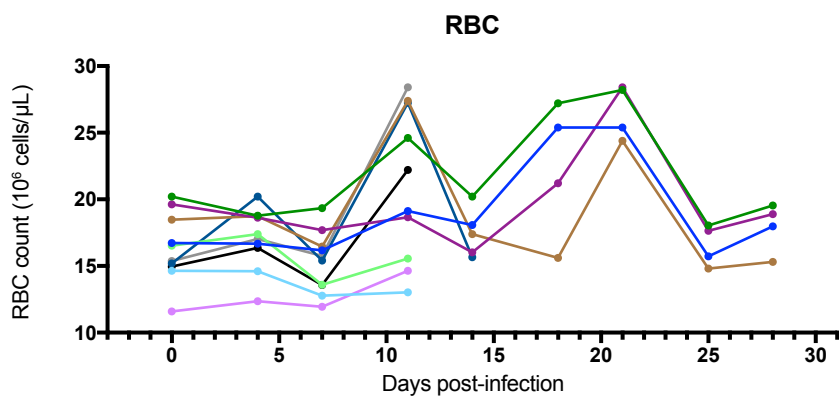

C

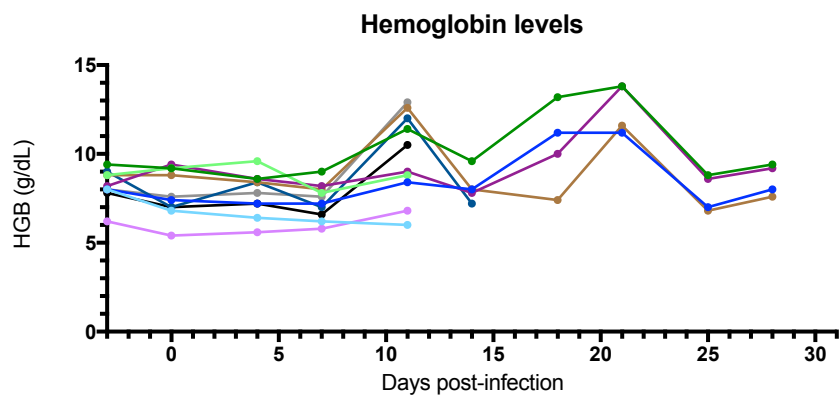

D

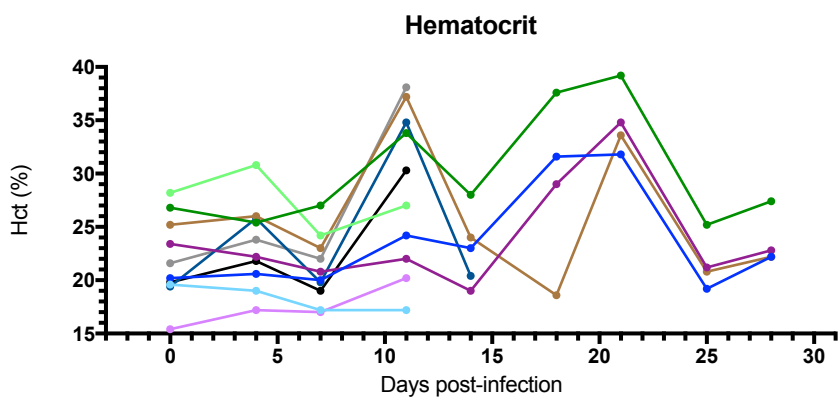

Supplement: Supplementary file 4 — Additional file 4. Haematological parameters measured from individual Mccp-infected animals. A) white blood cell count (103/μL), B) red blood cell count (106/μL), C) haemoglobin levels (g/dl) and D) haematocrit (%). [file 13567_2019_628_MOESM4_ESM.pdf]

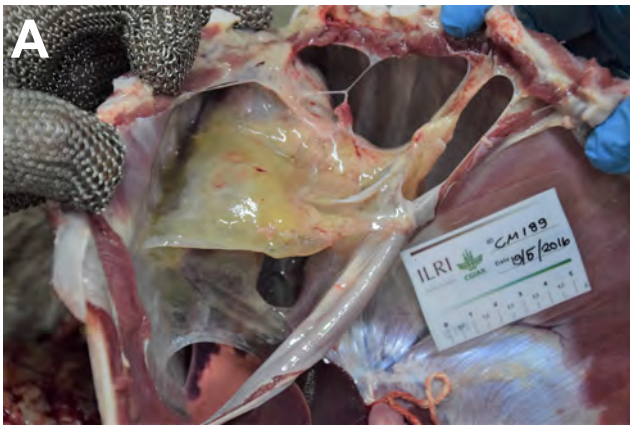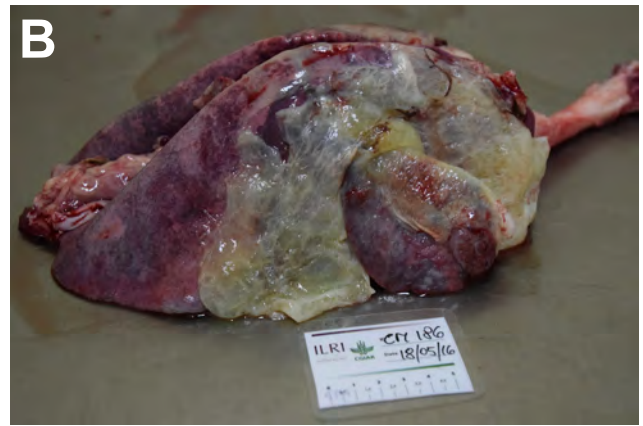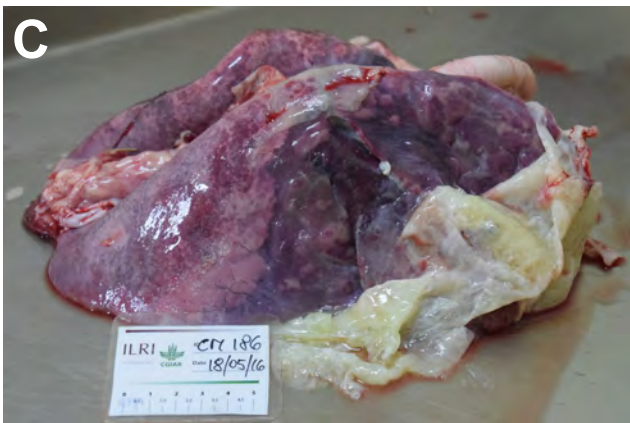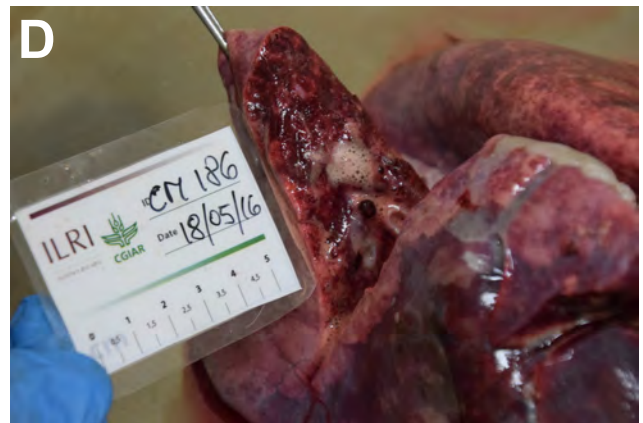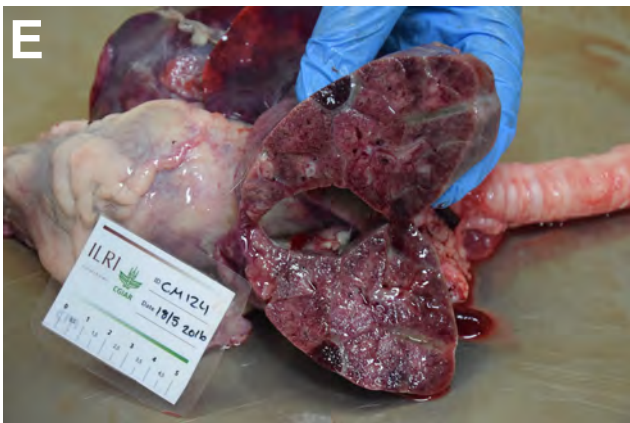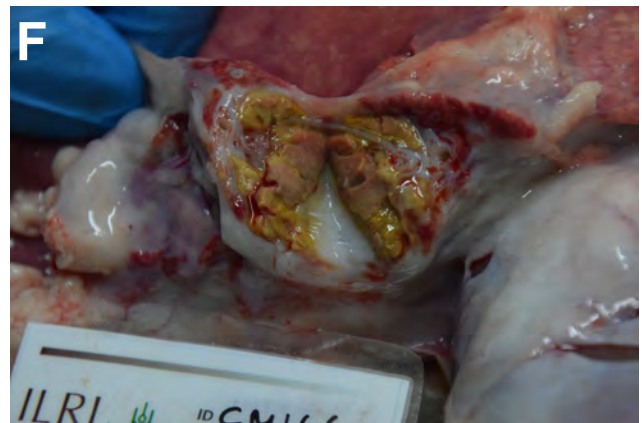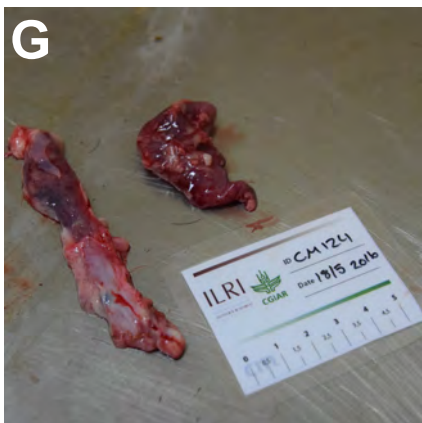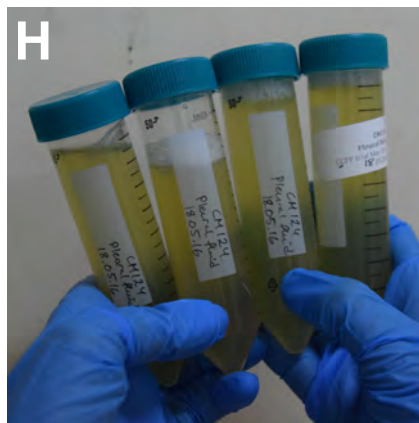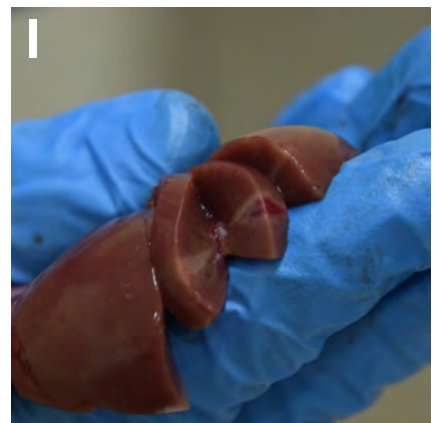

Supplement: Supplementary file 5 — Additional file 5. Macroscopic lesions observed during post-mortem. A: thoracic cavity with yellow fibrin accumulated between the parietal and visceral pleura in the right lung, creating fibrinous adhesions between the lung and the chest wall; B: lung showing fibrinous pleuropneumonia with extended deposits of fibrin covering the pulmonary (right) surface; C: pneumonia affecting more than 60% of the right lung parenchyma (apical lobe, medium lobe and cranial part of diaphragmatic lobe); D: transverse section of lung, acute inflammation of the parenchyma with congestion and pulmonary edema; E: cut section of lung lobe with pneumonia. Parenchyma is firm showing areas varying from acute inflammation (reddish) to necrosis (grayish); F: lung sequestra; necrotic tissue is surrounded by a white fibrotic capsule; G: enlarged and hemorrhagic respiratory lymph nodes (mediastinal and peribronchial); H: fibrinous pleural exudate collected from the thorax cavity of a single goat; I: kidney infarct, hemorrhagic area is surrounded by a pale white zone. [file 13567_2019_628_MOESM5_ESM.pdf]

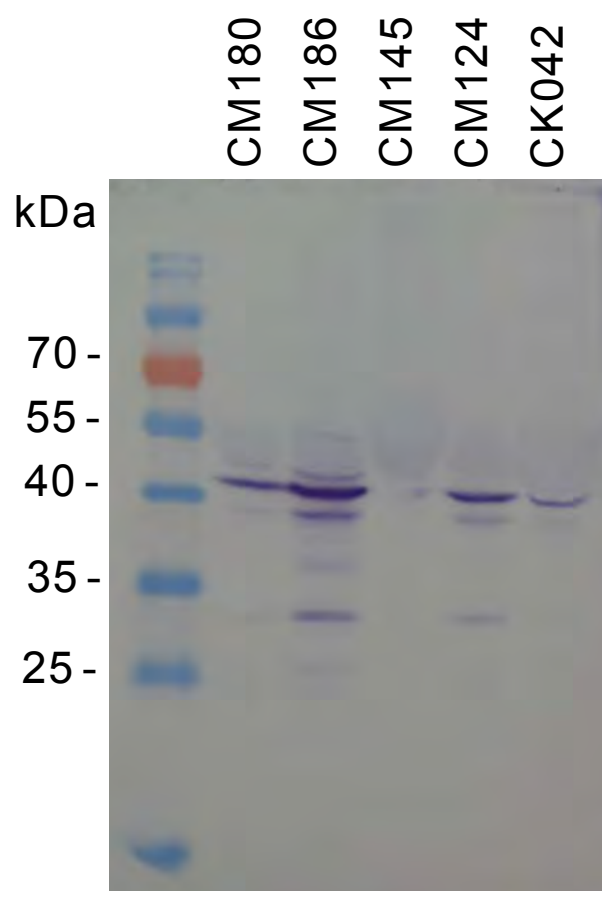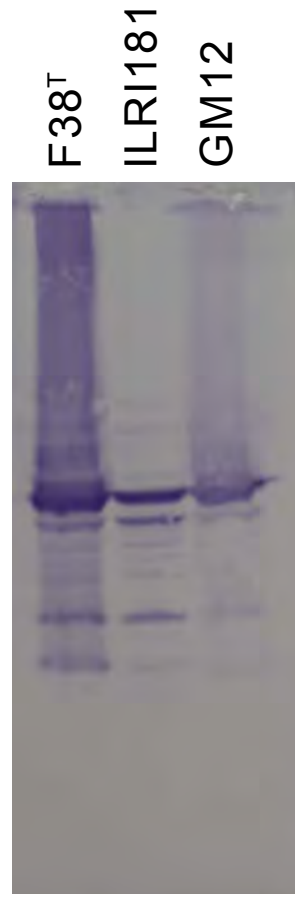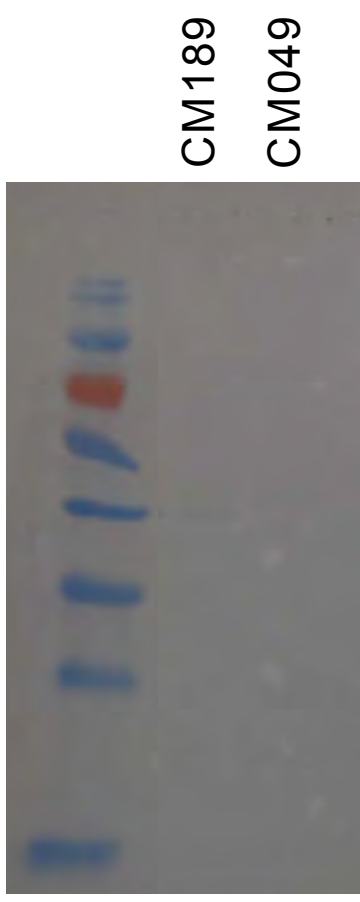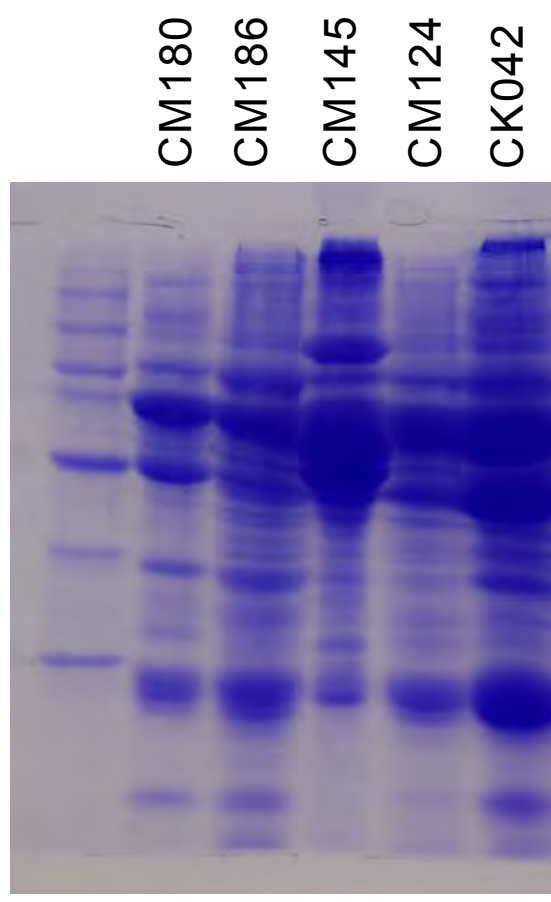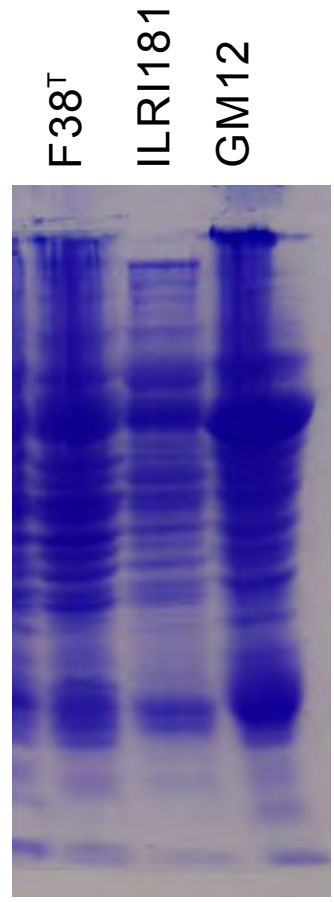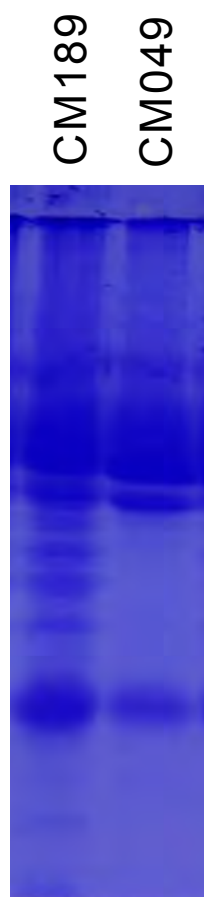

Supplement: Supplementary file 7 — Additional file 7. In vivo detection of glycerol-3-phospate oxidase (GlpO, 42.6 kDa). Pleural fluid samples were separated by SDS-PAGE and were transferred to nitrocellulose membranes for subsequent immunoblot analysis using a polyclonal rabbit anti-GlpO antibody. The right side contains the loading control of the samples that have been used in the immunoblot on the left side (Coomassie stain). Positive controls were the strains Mycoplasma capricolum subsp. capripneumoniae F38T and ILRI181 as well as Mycoplasma mycoides subsp. capri GM12 all expressing GlpO, negative controls were pleural fluids from animals CM189 and CM049 that contained very few Mycoplasma. [file 13567_2019_628_MOESM7_ESM.pdf]
