# Supplementary material for: Reproduction of contagious caprine pleuropneumonia reveals the ability of convalescent sera to reduce hydrogen peroxide production in vitro
Source: Vet Res. 2019 Feb 8;50:10. doi: 10.1186/s13567-019-0628-0 (PMC6368817; doi:10.1186/s13567-019-0628-0)
Supplement: Supplementary file 6 — Additional file 6. Serological responses against Mycoplasma capricolum subsp. capripneumoniae measured by IDEXX CCPP Ab Test. A) Table displaying individual serological responses of Mccp-infected animals. B) Antibody response (mean values) of euthanized vs surviving goats within the Mccp-infected group. C) Table displaying individual serological responses of mock-infected animals. All values are expressed as % of inhibition. Cutoff = 55%. Positive ≥ 55%. [file 13567_2019_628_MOESM6_ESM.pdf]

A

	Animal ID									
Days post infection	CK042	CM043	CM048	CM049	CM124	CM145	CM166	CM180	CM186	CM189
-2	23	20	32	12	18	26	21	5	16	22
0	28	29	30	19	27	39	22	21	30	32
4	32	36	23	23	25	35	13	25	29	33
7	36	49	27	17	26	37	26	54	32	34
11	32	**71**	41	52	32	47	44	52	53	42
14		**75**	**63**			**60**	**68**	41		
18		**77**	**79**			**84**	**71**			
21		**84**	**83**			**85**	**78**			
25		**87**	**83**			**84**	**84**			
28		**89**	**88**			**88**	**79**			
31		**80**	**79**			**77**	**73**			

B

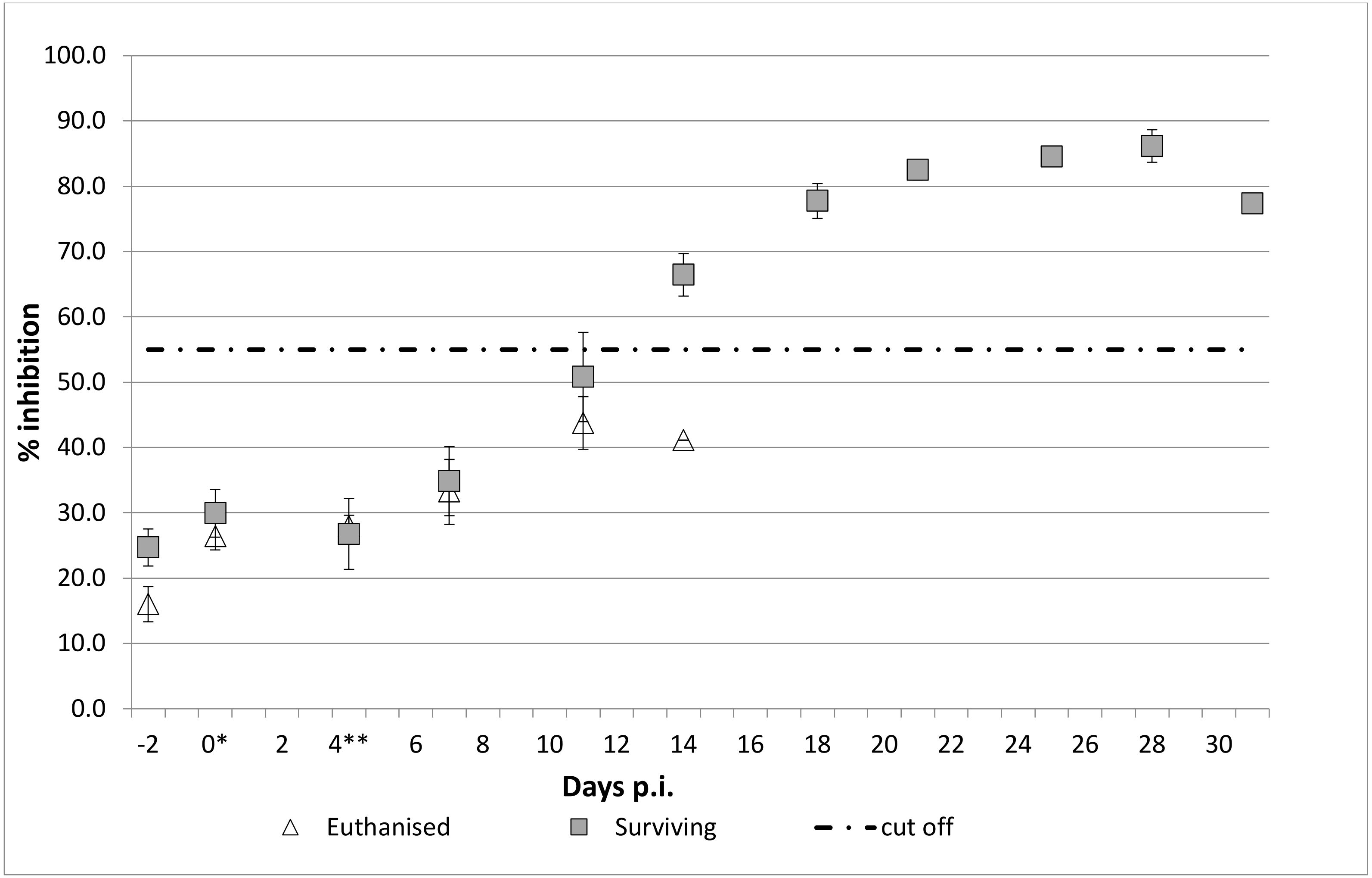

100.0
90.0
80.0
70.0
60.0
50.0
40.0
30.0
20.0
10.0
0.0
% inhibition
-2
0*
2
4**
6
8
10
12
14
16
18
20
22
24
26
28
30
Days p.i.
Euthanised
Surviving
cut off

C

	Animal ID				
Days post infection	CM233	CM251	CM253	CM260	CM261
-3	37	50	37	33	44
1	31	47	36	30	50
4	42	48	27	27	47
8	34	51	30	36	38
11	30	42	36	33	51
15	42	47	44	33	49
18	38	43	37	36	54
22	18	25	27	23	32
25	29	25	31	25	28
29	26	26	28	29	44
32	18	38	27	20	30
35	40	34	35	33	42
