# Supplementary material for: Reproduction of contagious caprine pleuropneumonia reveals the ability of convalescent sera to reduce hydrogen peroxide production in vitro
Source: Vet Res. 2019 Feb 8;50:10. doi: 10.1186/s13567-019-0628-0 (PMC6368817; doi:10.1186/s13567-019-0628-0)
Supplement: Supplementary file 8 — Additional file 8. Summary table of urine pH taken at necropsy. [file 13567_2019_628_MOESM8_ESM.pdf]

Animal ID	pH of urine
CK042[†]	6.4
CM043	8.4
CM048	8.5
CM049[†]	6
CM124[†]	6.7
CM145	8.5
CM166	8.9
CM180[†]	6.4
CM186[†]	6.4
CM189[†]	6.7
CM233*	ND
CM251*	ND
CM253*	8.5
CM260*	8.2
CM261*	8.5

[†]Euthanized before 31 dpi; *mock-infected animal; ND-not determined
